# CFTR negatively reprograms Th2 cell responses, and CFTR potentiation restrains allergic airway inflammation

**DOI:** 10.1172/jci.insight.191098

**Published:** 2025-03-25

**Authors:** Mark Rusznak, Christopher M. Thomas, Jian Zhang, Shinji Toki, Weisong Zhou, Masako Abney, Danielle M. Yanda, Allison E. Norlander, Craig A. Hodges, Dawn C. Newcomb, Mark H. Kaplan, R. Stokes Peebles, Daniel P. Cook

**Affiliations:** 1 Department of Internal Medicine, Vanderbilt University Medical Center, Nashville, Tennessee, USA.; 2 Department of Internal Medicine, University of Iowa, Iowa City, Iowa, USA.; 3 Department of Anatomy, Cell Biology, and Physiology, Indiana University School of Medicine, Indianapolis, Indiana, USA.; 4 Department of Genetics and Genome Sciences, Case Western Reserve University School of Medicine, Cleveland, Ohio, USA.; 5 Department of Pathology, Microbiology, and Immunology, Vanderbilt University Medical Center, Nashville, Tennessee, USA.; 6 Department of Microbiology and Immunology, Indiana University School of Medicine, Indianapolis, Indiana, USA.; 7 Tennessee Valley Healthcare System, U.S. Department of Veterans Affairs, Nashville, Tennessee, USA.; 8 Immunology Graduate Program, University of Iowa, Iowa City, Iowa, USA.

**Keywords:** Immunology, Inflammation, Pulmonology, Adaptive immunity, T cell development, Th2 response

## Abstract

Type 2 inflammatory diseases, including asthma, sinusitis, and allergic bronchopulmonary aspergillosis, are common in cystic fibrosis (CF). CD4^+^ Th2 cells promote these diseases through secretion of IL-4, IL-5, and IL-13. Whether the CF transmembrane conductance regulator (CFTR), the mutated protein in CF, has a direct effect on Th2 development is unknown. Using murine models of CFTR deficiency and human CD4^+^ T cells, we show that CD4^+^ T cells expressed *Cftr* transcript and CFTR protein following activation. Loss of T cell CFTR expression increased Th2 cytokine production compared with control cells. Mice with CFTR-deficient T cells developed increased allergic airway disease to *Alternaria alternata* extract compared with control mice. Culture of CFTR-deficient Th2 cells demonstrated increased IL-4Rα expression and increased sensitivity to IL-4 with greater induction of GATA3 and IL-13 compared with control Th2 cell cultures. The CFTR potentiator ivacaftor reduced allergic inflammation and type 2 cytokine secretion in bronchoalveolar lavage of humanized CFTR mice following *Alternaria alternata* extract challenge and decreased Th2 development in human T cell culture. These data support a direct role of CFTR in regulating T cell sensitivity to IL-4 and demonstrate a potential CFTR-specific therapeutic strategy for Th2 cell–mediated allergic disease.

## Abstract 2

CFTR deficiency in T cells enhances Th2 differentiation and effector function by augmenting IL-4 sensitivity. CFTR potentiation reduces allergic inflammation, offering therapeutic potential for allergy.

## Graphical abstract

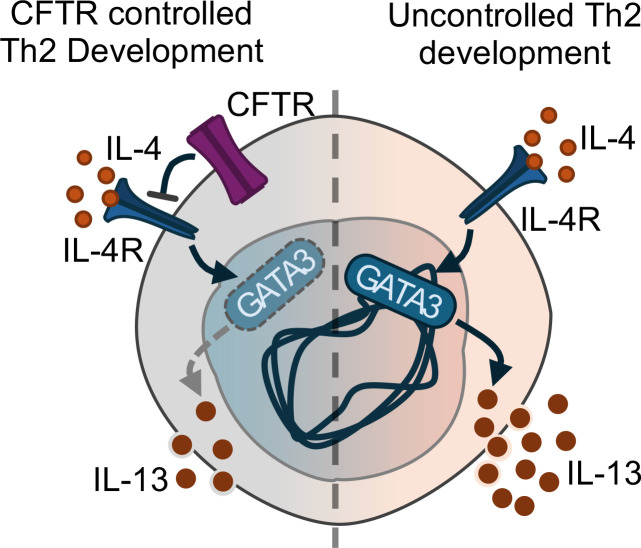

## Introduction

Cystic fibrosis (CF) is a common autosomal recessive disease caused by mutations of the gene encoding a chloride/bicarbonate channel known as the CF transmembrane conductance regulator (CFTR). CF is characterized by significant pulmonary morbidity and mortality as a result of recurrent inflammation and mucus accumulation in the lungs. While the main CF airway pathologic feature is severe neutrophilic inflammation, recent studies have suggested a role for type 2 inflammation, in part driven by the adaptive immune system via CD4^+^ Th2 cells activated by chronic allergen exposure (1–5). Activated Th2 effector cells produce type 2 cytokines including IL-4, IL-5, and IL-13. IL-4 and IL-13 promote the differentiation of naive CD4^+^ cells toward Th2 development, B cell class switching to immunoglobin E (IgE) production, and goblet cell hyperplasia. IL-5 serves as a major recruitment, differentiation, and survival factor for eosinophils. All 3 ILs contribute to type 2 inflammation and have been implicated in CF immune dysregulation (2, 6–10).

Studies of persons with CF and animal models deficient of CFTR activity have shown an elevated IgE response to fungal allergens (10–13), with increased levels of IL-4 and IL-13 compared with normal CFTR function (7, 10, 13, 14). Naive CD4^+^ T cells from *Cftr*-deficient mice had elevated IL-4 production after TCR ligation (13) and increased polarization and effector function compared with CD4^+^ T cells from *Cftr^+/+^* controls (10, 15). In patients with CF, type 2 inflammation was a prognostic factor for *Pseudomonas* infection, and a type 2 inflammatory phenotype was correlated with increased risk of infection, pulmonary exacerbation independent of bacterial colonization, and death (3, 4, 16). In our recent studies, *Cftr^−/−^* mice demonstrated an elevated type 2 inflammatory response to the ubiquitous aeroallergen, *Alternaria alternata*, compared with *Cftr^+/+^* controls (15). In these adaptive model studies, loss of CFTR led to increased cytokine levels of IL-5 and IL-13 in the bronchoalveolar lavage fluid (BALF), greater recruitment of lymphocytes and eosinophils to the airway, and elevations in serum total IgE compared with CFTR-sufficient controls. In support of a CD4^+^ T cell–specific effect, cultured Th2 cells from *Cftr^−/−^* mice in the presence of IL-33 had increased IL-13 secretion compared with similarly cultured *Cftr^+/+^* controls (15), suggesting a direct function of CFTR in inhibiting Th2 development. Despite these prior studies, whether CFTR intrinsically and negatively regulates Th2 cell function, as well as the effects of CFTR-targeted therapy in CD4^+^ T cells, remains poorly understood.

The goal of this study was to determine whether CFTR negatively regulates CD4^+^ T cell differentiation and effector function in allergic disease and if strategies to increase CFTR function may provide therapeutic potential in airway inflammation through T cell direct and indirect mechanisms. To test this hypothesis, we aimed to (a) assess T cell–specific CFTR loss in in vitro and in vivo models of allergic airway inflammation, (b) determine the molecular pathways underlying increased CD4^+^ T cell effector function in response to CFTR functional loss, and (c) test for the therapeutic benefit of CFTR potentiation in Th2-mediated allergic disease.

## Results

### CFTR is expressed in CD4^+^ T cells and induced with T cell activation.

**Figure 1:**
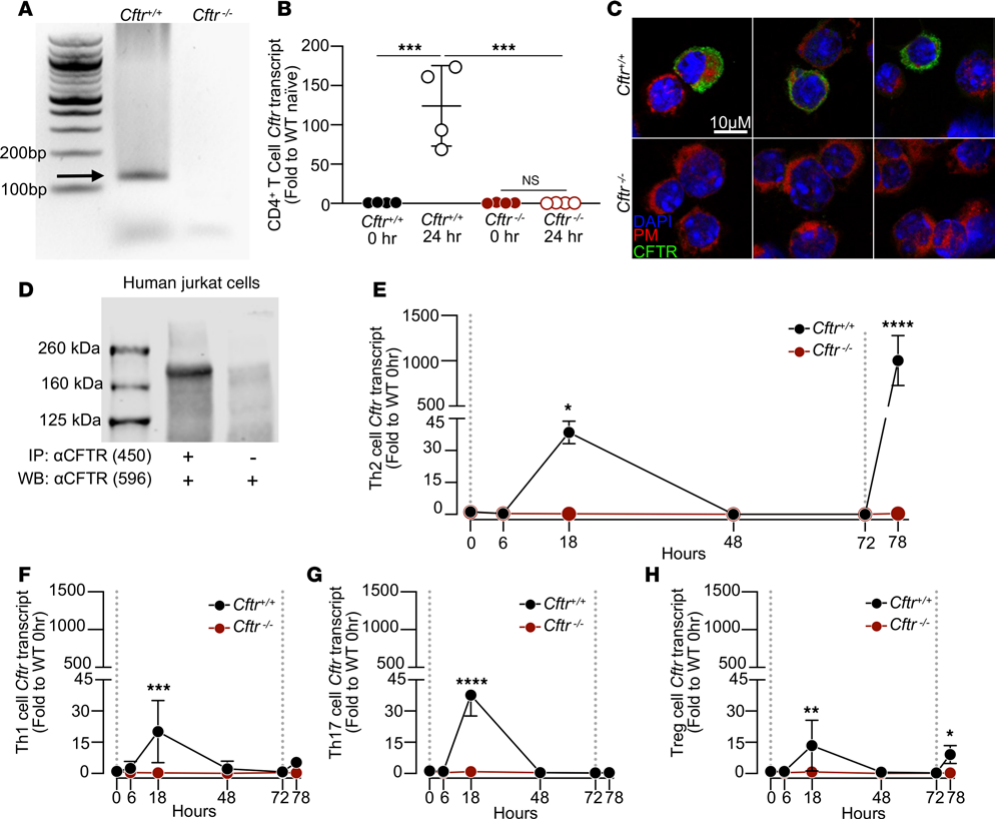
CFTR is expressed in CD4^+^ T cells, induced with CD4^+^ T cell polarization, and upregulated with Th2 cell reactivation. (**A**) Reverse transcriptase PCR gel for *CFTR mRNA* (predicted size of 125 bp; arrow) in *Cftr^+/+^ and Cftr^−/−^* mouse CD4^+^ T cells. Lane 1 is a 100 bp ladder. (**B**) qPCR analysis of *Cftr* expression in *Cftr^+/+^* and *Cftr^−/−^* mouse CD4^+^ T cells at 0 and 24 hours following TCR ligation with anti-CD3 and anti-CD28 mAbs (*n* = 4 per genotype and time point). (**C**) Immunostaining of CFTR (green), plasma membrane lectin (red), and nuclei (blue) in 24-hour–cultured mouse CD4^+^ cells (3 different biologic replicates per genotype, *Cftr^+/+^* and *Cftr^−/−^*). Scale bar: 10 μm. (**D**) Immunoprecipitated CFTR protein in immortalized human CD4^+^ T cells (Jurkat) using UNC-450 anti-CFTR monoclonal antibody (mAb) for pulldown and UNC-596 anti-CFTR mAb for detection compared with immunoprecipitation isotype control. (**E**–**H**) qPCR analysis of *Cftr* expression in *Cftr^+/+^* and *Cftr^−/−^* cultured mouse CD4^+^ T cells at 0, 6, 18, 48, 72, and 78 hours in Th2 (**E**), Th1 (**F**), Th17 (**G**), and Tregs (**H**) (*n* = 3 per genotype per time point). Dotted lines represent TCR ligation with anti-CD3 (1 μg/mL) and anti-CD28 (0.5 μg/mL) mAbs and (a) anti–IFN-γ (10 μg/mL) and mouse IL-4 (10 ng/mL) for Th2 cells, (b) anti–IL-4 (10 μg/mL) and mouse IL-12 (10 ng/mL) for Th1 cells, and (c) human TGF-β (0.5 ng/mL), mouse IL-23 (10 ng/mL), mouse IL-6 (40 ng/mL), mouse IL-1b (10 ng/mL), anti–IL-4 (10 μg/mL), and anti–IFN-γ (10 μg/mL) for Th17 cells. Tregs were polarized and restimulated with anti-CD3 (1 μg/mL), human IL-2 (100 IU/mL), and recombinant human TGF-β (1ng/mL). Data are shown as mean ± SD. Statistical analysis were done in **B** and **E**–**H** by 1-way ANOVA followed by Tukey’s honestly significant difference (HSD) post hoc test for multiple comparisons. *P < 0.05, **P < 0.01, ***P < 0.001, and ****P < 0.0001.

To investigate whether mouse CD4^+^ T cells express CFTR, we isolated RNA and performed reverse transcriptase PCR. *Cftr* mRNA was present in freshly isolated CD4^+^ T cells from splenocytes from *Cftr^+/+^* mice but was absent in CD4^+^ T cells from *Cftr^−/−^* mice (Figure 1A and Supplemental Figure 1; supplemental material available online with this article; https://doi.org/10.1172/jci.insight.191098DS1). CD62L^hi^CD44^lo^CD4^+^ (naive) T cells were isolated and either processed for RNA or cultured with anti-CD3 (1 μg/mL) and anti-CD28 (0.5 μg/mL) for 24 hours (Supplemental Figure 2). The resulting RNA was then used to perform quantitative PCR (qPCR) to determine the relative abundance of *Cftr* transcripts in *Cftr^+/+^* and *Cftr^−/−^* mouse CD4^+^ T cells at 0 hours (naive) and 24 hours after activation (Figure 1B). When normalized to naive *Cftr^+/+^* CD4^+^ T cells, *Cftr^+/+^* CD4^+^ T cell activation resulted in a significant increase in *Cftr* transcripts, an effect not seen in activated *Cftr*^−/−^ CD4^+^ T cells. Immunostaining showed that CFTR primarily localized to both surface and intracellular compartments in a subset of cultured and activated *Cftr^+/+^* CD4^+^ T cells at 24 hours (Figure 1C). CFTR was not detected in cultured CD4^+^ T cells from *Cftr^−/−^* mice. To determine whether CFTR expression occurs in human CD4^+^ T cells, cell lysates from an immortalized cell line of T lymphocyte cells (Jurkat) were immunoprecipitated with an antibody directed to the R domain of CFTR (UNC-450) or isotype (IgG_1_) control and immunoblotted with an antibody directed to the second nucleotide binding domain of CFTR (UNC-596). Immunoblot of the immunoprecipitated Jurkat cellular lysates revealed the presence of an approximately 170 kDa band consistent with CFTR in the CFTR immunoprecipitated samples but not in the isotype control samples (Figure 1D).

To understand the temporal kinetics of *Cftr* expression following activation and/or polarization of the CD4^+^ T cell, we next measured relative *Cftr* transcript abundance following activation with anti-CD3 (1 μg/mL) and anti-CD28 (0.5 μg/mL) and polarization toward Th2, Th1, and Th17 cell subsets (Figure 1, E–G), with stimulation as follows: Th2, anti–IFN-γ (10 μg/mL) and mouse IL-4 (10 ng/mL); Th1, anti–IL-4 (10 μg/mL) and mouse IL-12 (10 ng/mL); and Th17, human TGF-β (0.5 ng/mL), mouse IL-23 (10 ng/mL), mouse IL-6 (40 ng/mL), mouse IL-1β (10 ng/mL), anti–IL-4 (10 μg/mL), and anti–IFN-γ (10 μg/mL). Tregs were polarized and restimulated with anti-CD3 (1 μg/mL), human IL-2 (100 IU/mL), and recombinant human TGF-β (0.5 ng/mL) (Figure 1H). In all 4 subsets, a significant increase in *Cftr* transcript was noted by 18 hours, with the largest increases in Th2 and Th17 subsets. By 48 hours, *Cftr* transcript levels returned to undetectable levels in all 4 subsets. Restimulation with respective activation/polarization cytokines and monoclonal antibodies (mAbs) significantly induced *Cftr* transcripts to a small level in Tregs (Figure 1H), but there were significantly increased *Cftr* transcripts in Th2 cells nearly 20 times the amount at the 18 hour time point (Figure 1E). Taken together, these results demonstrate that CFTR is expressed in CD4^+^ T cells, *Cftr* is induced with activation, and Th2 cells display the greatest amounts of *Cftr* transcript changes with restimulation.

### CFTR negatively regulated Th2 effector function.

**Figure 2:**
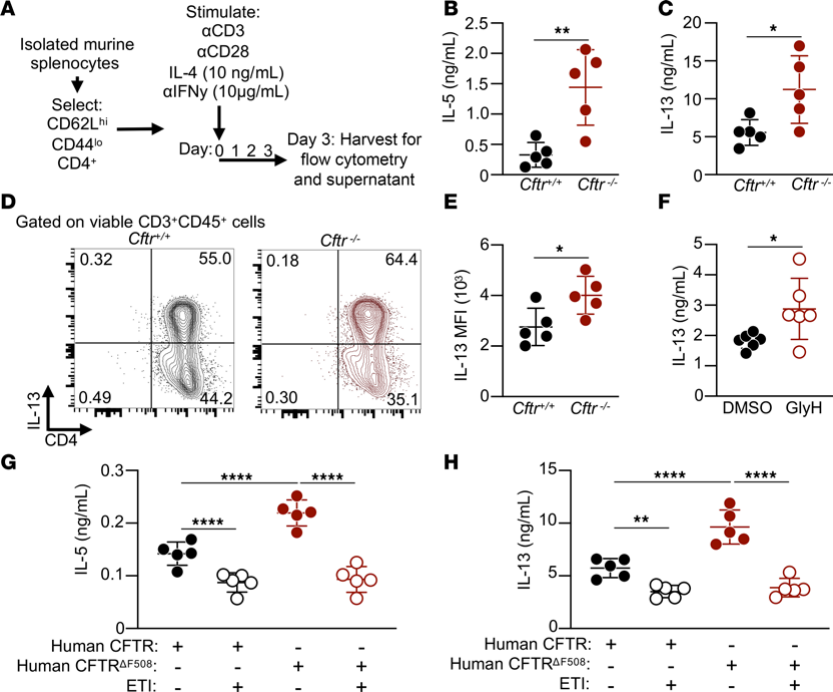
Loss of CFTR increases Th2 polarization and effector function. (**A**) Schematic diagram showing isolation and stimulation of naive CD4^+^ T cells. (**B** and **C**) IL-5 and IL-13 by ELISA in cellular supernatant from *Cftr*^+/+^ and *Cftr*^−/−^ *CD4*^+^ T cells grown in culture stimulated with mouse IL-4 (*n* = 5 mice per genotype). (**D**) Representative gating strategy for IL-13 expression in cultured *Cftr*^+/+^ and *Cftr*^−/−^ *CD4*^+^ T cell populations gated on live lymphoid cells. (**E**) IL-13 median fluorescence intensity (MFI) of cultured *Cftr*^+/+^ and *Cftr*^−/−^ *CD4*^+^ T cells (*n* = 5 mice per genotype). (**F**) IL-13 by ELISA in cellular supernatant from *Cftr*^+/+^ CD4^+^ T cells grown in culture with the CFTR inhibitor, GlyH-101, or control vehicle (DMSO) stimulated with mouse IL-4. (**G** and **H**) IL-5 and IL-13 by ELISA in cellular supernatant from mouse CD4^+^ T cells expressing either wild-type human CFTR or *hCFTR*^ΔF508^ grown in culture with Elexacaftor/Tezacaftor/ Ivacaftor (ETI) or DMSO control (*n* = 5 mice per genotype per condition). Data are shown as mean ± SD. Statistical analysis were performed using unpaired Student’s *t* test (**B**, **C**, **E**, and **F**) and by 1-way ANOVA (**G** and **H**) followed by Tukey’s honestly significant difference (HSD) post hoc test for multiple comparisons. *P < 0.05, **P < 0.01, and ****P < 0.0001.

Given our previously published studies demonstrating increased type 2 inflammation with loss of CFTR and our data showing that Th2 cells induce *Cftr* transcript following activation and polarization, we hypothesized that CFTR functions as a negative regulator of Th2 cell function. To test this hypothesis, we isolated naive T cells from *Cftr^+/+^* and *Cftr^−/−^* mouse spleens, provided TCR ligation with anti-CD3 (1 μg/mL) and anti-CD28 (0.5 μg/mL), and polarized with mouse IL-4 (10 ng/mL) and anti–IFN-γ (10 μg/mL) for 3 days prior to flow cytometry and cellular supernatant analysis (Figure 2A). After 3 days of culture in these conditions, *Cftr^−/−^* CD4^+^ T cells secreted significantly more IL-5 (Figure 2B) and IL-13 (Figure 2C) as measured in the cellular supernatant compared with *Cftr^+/+^* CD4^+^ T cells. Single-cell analysis of cultured cells by flow cytometry revealed a larger percentage of IL-13^+^CD4^+^ T cells (Figure 2D) and a higher median fluorescence intensity (MFI) of IL-13 in *Cftr*^−/−^ CD4^+^ T cells compared with *Cftr*^+/+^ CD4^+^ T cells (Figure 2E). No differences were observed in proliferative capacity of *Cftr*^−/−^ CD4^+^ T cells compared with *Cftr*^+/+^ CD4^+^ T cells (Supplemental Figure 3, A and B). Intracellular IL-4 was expressed in a higher percentage but on a per-cell basis to a similar amount based on MFI in *Cftr*^−/−^ CD4^+^ T cells compared with *Cftr*^+/+^ CD4^+^ T cells (Supplemental Figure 4, A–C). Th1- and Th17-polarized *Cftr*^−/−^ CD4^+^ T cells secreted similar amounts of IFN-γ and IL-17, respectively, compared with *Cftr^+/+^* CD4^+^ T cells, while Treg-polarized *Cftr*^−/−^ CD4^+^ T cells secreted increased IL-10 compared with *Cftr^+/+^* CD4^+^ T cells (Supplemental Figure 5, A–C).

To limit the possible confounding of systemic and chronic CFTR loss toward intrinsic Th2 skewing and to test whether acute loss of CFTR would increase CD4^+^ T cell cytokine production, *Cftr^+/+^* CD4^+^ T cells were cultured under similar Th2 polarizing conditions in the presence of the CFTR inhibitor GlyH-101 (500 nM) or DMSO (1% v/v) control for 3 days. Compared with DMSO treated *Cftr^+/+^* CD4^+^ T cells, acute inhibition of CFTR by GlyH-101 significantly increased the amount of secreted IL-13 (Figure 2F). Since we observed increased Th2 effector function in CD4^+^ T cells without functional CFTR in KO and pharmacological inhibition models, we next hypothesized that increased CFTR function reduces Th2 effector function. To test this, we utilized the approved CFTR potentiator ivacaftor (1 μM) to increase the open probability of CFTR channels, and the CFTR correctors elexacaftor (3 μM) and tezacaftor (3 μM, elexacaftor-tezacaftor-ivacaftor [ETI]) to augment CFTR folding in a recently developed “humanized CFTR” mouse model (17, 18). This mouse model — which was generated in a *Cftr*-null background with a bacterial artificial chromosome transgene carrying the complete human *CFTR* (*hCFTR*) gene, including its regulatory elements — was critical for these experiments as the CFTR potentiator ivacaftor is unable to potentiate mouse CFTR (19, 20). Conversely, ivacaftor is capable of potentiating mutated and WT hCFTR (21). CD4^+^ T cells isolated from mice deficient in mouse CFTR (*Cftr^−/−^*) but possessing the *hCFTR* transgene and similar mice possessing a mutation in the *hCFTR* gene resulting in a deletion of phenylalanine 508 (*hCFTR^ΔF508^*) were cultured in Th2 polarizing conditions with either ETI or DMSO (control). Mutated *hCFTR^ΔF508^* CD4^+^ T cells had significantly increased IL-5 (Figure 2G) and IL-13 (Figure 2H) secretion in the cellular supernatant compared with *hCFTR* CD4^+^ T cells in control conditions. ETI significantly reduced IL-5 and IL-13 secretion in both genotypes. No differences were observed in the proliferative capacity of ETI-treated *hCFTR* CD4^+^ T cells compared with DMSO-treated *hCFTR* CD4^+^ T cells (Supplemental Figure 6, A and B). These results demonstrate that loss of CFTR function increased Th2 effector function and that, conversely, augmentation of CFTR function significantly reduced CD4^+^ T cell secretion of type 2 cytokines.

### T cell–specific loss of CFTR increases allergic inflammation.

**Figure 3:**
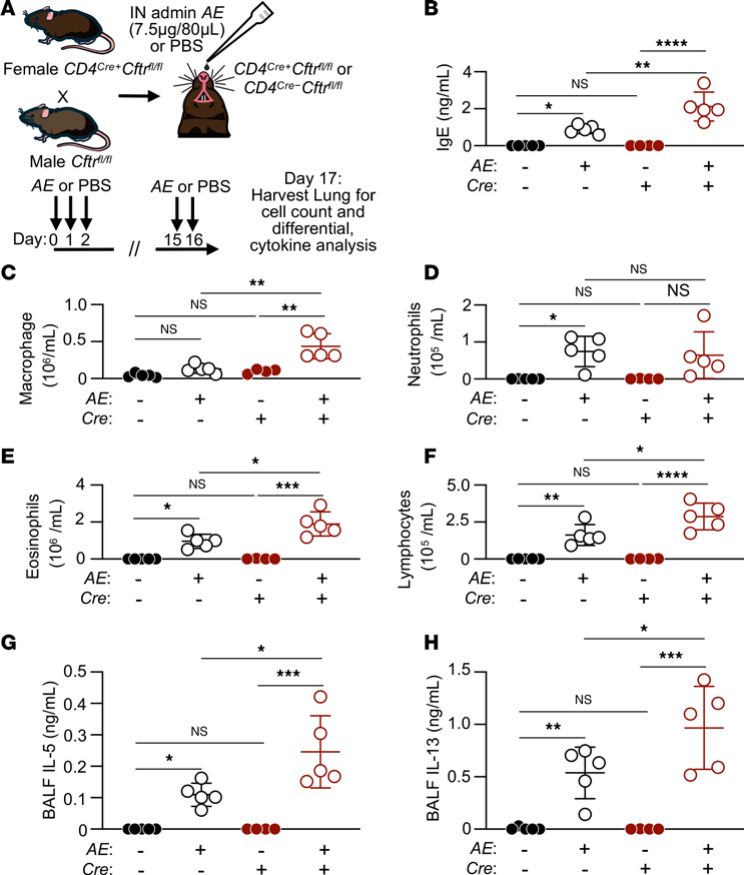
CD4^+^ T cell–specific CFTR deficiency increases *Alternaria* extract–induced (*AE*-induced) allergic inflammation. (**A**) Schematic diagram showing adaptive model of intranasal *AE*-induced airway inflammation in *CD4^Cre−^Cftr^fl/fl^* and *CD4^Cre+^Cftr^fl/fl^* mice. (**B**) IgE concentrations by ELISA in serum from treated mice (*n* = 4–5 depending on genotype and condition). (**C**–**F**) The number of macrophages (**C**), neutrophils (**D**), eosinophils (**E**), and lymphocytes (**F**) in the BALF of PBS- or *AE*-challenged mice (*n* = 4–5 per genotype and condition). (**G** and **H**) IL-5 and IL-13 by ELISA in BAL from *CD4^Cre−^Cftr^fl/fl^* and *CD4^Cre+^Cftr^fl/fl^* mice treated with either *AE* or PBS control (*n* = 4–5 per genotype and condition). Open circles represent *AE* sensitized and challenged mice, and closed circles denote PBS control mice. Black circles denote *CD4^Cre−^Cftr^fl/fl^* and red circles indicate *CD4^Cre+^Cftr^fl/fl^* mice. Statistical analysis in **B**–**H** were done by 1-way ANOVA followed by Tukey’s honestly significant difference (HSD) post hoc test for multiple comparisons. *P < 0.05, **P < 0.01, ***P < 0.001, and ****P < 0.0001.

To test whether T cell–specific loss of CFTR increases allergic inflammation to *Alternaria* extract (AE), we created a mouse T cell–specific *Cftr*-KO model by crossing previously described *Cftr^fl/fl^* mice (22) to CD4^Cre+^ mice (23), specifically deleting CFTR during T cell thymic development. To test the role of CFTR in T cells during allergic inflammation, *CD4^Cre+^Cftr^fl/fl^* and *CD4^Cre−^Cftr^fl/fl^* littermate mice were sensitized and challenged with either *AE* (7.5 μg) or PBS control using an adaptive model of allergic airway inflammation (Figure 3A). Extract of *Alternaria*, a ubiquitous fungal aeroallergen, was used as the airway antigen challenge, as it is a common fungal sensitizing antigen in CF (24), is associated with strong bronchial provocations in CF (25), and elicits a strong type 2 airway immune response, greater in *Cftr* deficient compared with *Cftr* sufficient mice (15). To broadly assess the adaptive immune response in this model, serum total IgE was measured. IgE was significantly increased in *AE*-challenged *CD4^Cre−^Cftr^fl/fl^* mice compared with PBS-challenged mice, and *AE* challenge further significantly increased IgE in *CD4^Cre+^Cftr^fl/fl^* mice compared with *CD4^Cre−^Cftr^fl/fl^* mice (Figure 3B). To assess whether these serum IgE changes correlated with type 2 inflammatory cell airway recruitment, *AE*-induced cellular inflammation was assessed in the BALF (Figure 3, C–F). BALF cell differential counts revealed marked increases in inflammation in *AE*-challenged *CD4^Cre+^Cftr^fl/fl^* and *CD4^Cre−^Cftr^fl/fl^* mice compared with PBS challenge, with increases in eosinophils (Figure 3E) and lymphocytes (Figure 3F). T cell–specific loss of CFTR increased the overall type 2 inflammatory response, including significant elevations in recruited eosinophils and lymphocytes compared with *CD4^Cre−^Cftr^fl/fl^* controls (Figure 3, E and F). Cell type–specific percentage changes were only noted in comparison of PBS and *AE*-challenged BALF, demonstrating that *AE* induced a predominately eosinophilic response in both *CD4^Cre+^Cftr^fl/fl^* and *CD4^Cre−^Cftr^fl/fl^* mice (Supplemental Figure 7, A–D). Consistent with these findings, loss of CFTR in CD4^+^ T cells significantly increased IL-5 (Figure 3G) and IL-13 (Figure 3H) cytokine secretion measured in BALF of *CD4^Cre+^Cftr^fl/fl^* compared with *CD4^Cre−^Cftr^fl/fl^* mice. These data demonstrate that, in addition to previously characterized epithelial contributions to the exaggerated type 2 immune response seen in *Cftr*^−/−^ mice (15), the intrinsic loss of CFTR in CD4^+^ T cells increased Th2 effector cytokine release and augmented type 2 adaptive immune responses.

### Loss of CFTR in Th2 cells increased transcripts related to the IL-4/GATA3 axis.

**Figure 4:**
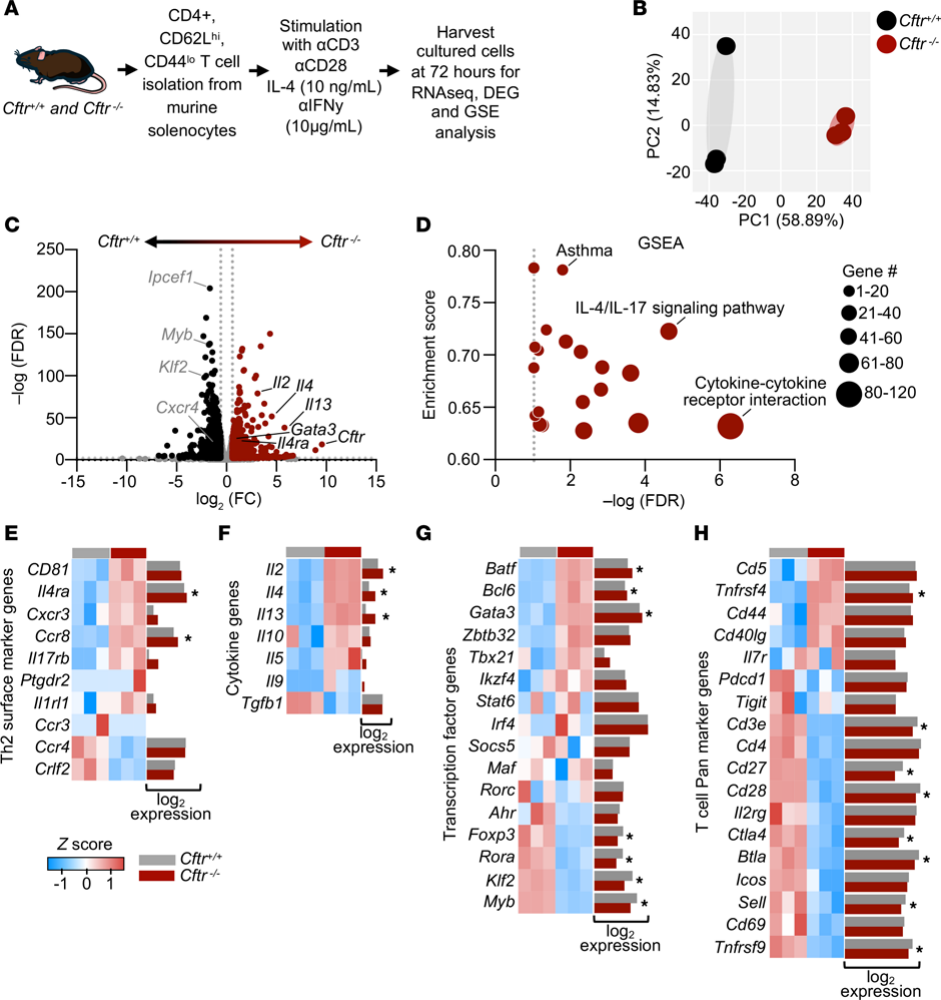
Loss of CFTR in Th2 cells increases whole transcriptome type 2 immune–specific gene expression. (**A**) Schematic diagram showing isolation and polarization of naive CD4^+^ T cells to Th2 cells for whole transcriptome analysis. (**B**) PCA plot of gene expression data for 3 biological replicates used for bulk RNA-Seq of *Cftr*^+/+^ (black dots) and Cftr^−/−^ (red dots) Th2 cells. (**C**) Volcano plot depicting DESeq2 analysis of differentially expressed genes in *Cftr*^+/+^ and *Cftr*^−/−^ Th2 cells. Red dots represent genes expressed at higher levels in *Cftr*^−/−^ Th2 cells, while black dots represent genes with higher expression levels in *Cftr*^+/+^ Th2 cells. The *y* axis denotes −log_10_ FDR values while the *x* axis shows log_2_ fold change values. Select genes indicated. (**D**) Advanced bubble plot showing KEGG pathways enriched in *Cftr*^−/−^ Th2 cells. The *y* axis denotes enrichment score, and −log_10_ FDR values are shown on the *x* axis. The size of the bubble represents the number of genes enriched in each pathway. Select pathways indicated. (**E**–**H**) Heatmaps showing the differential gene expression profile of core Th2 associated genes including surface markers (**E**), cytokines (**F**), transcription factors (**G**), and CD4^+^ pan markers (**H**) in *Cftr*^−/−^ versus *Cftr*^+/+^ Th2 cells. Normalized log_2_ gene expression determined by RNA-Seq shown to the right of each heatmap with statistically significant DEGs denoted (*) in *Cftr*^+/+^ (gray bars) and *Cftr*^−/−^ (red bars) Th2 cells. RNA-Seq data were generated in biological triplicates from 3 mice and analyzed via DESeq2. KEGG was used for pathway analysis.

We next used our mouse Th2 culture model to probe Th2 transcriptional changes due to CFTR loss. Naive CD4^+^ T cells were isolated and stimulated with anti-CD3, anti-CD28, IL-4, and anti–IFN-γ as described above (Figure 4A). On day 3 (72 hours) of culture, Th2 cells from 3 distinct biological mouse donors per genotype were assessed by RNA-Seq. Principal component analysis (PCA) demonstrated dissimilarity between the *Cftr^+/+^* and *Cftr^−/−^* Th2 cells (Figure 4B). Using differential expression genes (DEGs) analysis, 744 genes were differentially upregulated and 892 genes were downregulated in *Cftr^−/−^* Th2 cells compared with *Cftr^+/+^* Th2 cells (Figure 4C and Supplemental Tables 1 and 2). Supportive of a drive to transcribe *Cftr* mRNA in CD4^+^ T cells, increased levels of *Cftr* transcript were observed in *Cftr^−/−^* samples, as the model is an in-frame elimination of exon 11 and capable of producing nonfunctional alternative mRNA (26). Functional annotation using gene set enrichment analysis (GSEA) showed that upregulation of Th2 specific pathways including those related to asthma, IL-4/IL-17 signaling, and cytokine-cytokine receptor interactions (Figure 4D and Supplemental Table 3). A collection of T cell– and Th2-related gene signatures was generated using the Th2 cell gene set from Harmonizome multi-omics data integration platform (Ma’ayan Laboratory of Computational Systems Biology; ref. 27) including genes for surface markers (Figure 4E), cytokines (Figure 4F), transcription factors (Figure 4G), and pan markers (Figure 4H). Several regulators of canonical Th2 programming were increased by loss of CFTR in Th2 cells including *Il4ra*, *Il4*, *Il13*, and *Gata3*. Additional transcripts related to activation and/or exhaustion were dysregulated in *Cftr^−/−^* Th2 cells compared with *Cftr^+/+^* Th2 cells, including *Ccr8*, *Bcl6*, *Batf*, *Klf2*, *Myb*, *Trfrsf4* (encodes OX40), *Ctla4*, *Btla*, and *Tnfrsf9* (encodes 4-1BB). Gene concept networking revealed some of the most enriched nodal terms as JAK/STAT signaling, cytokine receptor signaling, and asthma gene sets (Supplemental Figure 8A). These 3 pathways demonstrated substantial overlap in a gene concept network, further supporting the role of CFTR in modifying type 2 inflammatory signaling. Hierarchical clustering of the 30 most enriched Gene Ontology: Biological Processes (GO:BP) terms showed alterations in anion transport, fatty acid metabolic processes, amide transport, and noncoding RNA metabolic processes (Supplemental Figure 8B). These broad categories of transcriptomic changes highlight the profound effect of CFTR loss on Th2 cells beyond canonical inflammatory pathways. Taken together, these data demonstrate that the loss of CFTR during activation and Th2 polarization fundamentally altered the Th2 transcriptome with specific skewing toward increased transcription of the IL-4/GATA3/IL-13 signaling axis.

### Loss of CFTR increased CD4^+^ T cell sensitivity to IL-4.

**Figure 5:**
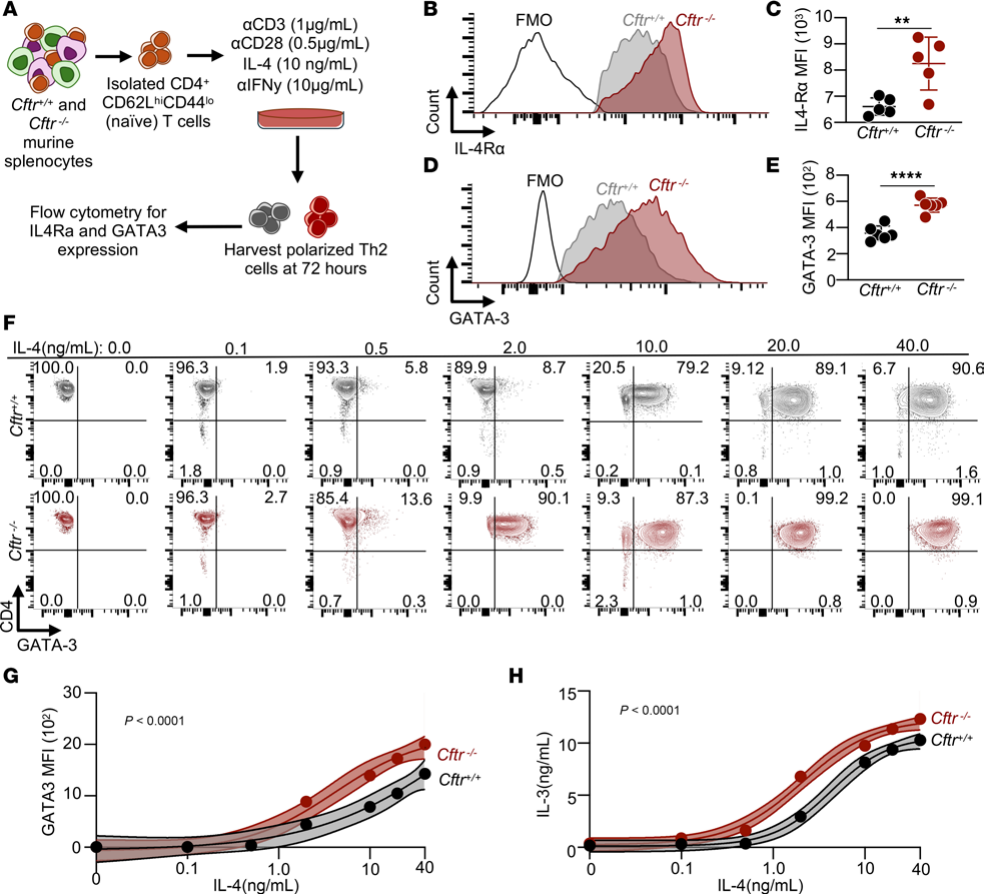
CFTR deficiency enhances IL-4 sensitivity and GATA3 expression in Th2 cells. (**A**) Schematic diagram showing isolation and polarization conditions of naive CD4^+^ T cells to Th2 cells for IL-4 studies. (**B**) Representative flow cytometry histogram showing the median fluorescence intensity (MFI) of IL-4Rα at 72 hours for *Cftr*^+/+^ and *Cftr*^−/−^ Th2 cells. (**C**) The quantified MFI of IL-4Rα in *Cftr*^+/+^ and *Cftr*^−/−^ Th2 cells at 72 hours (*n* = 5 mice per genotype). (**D**) Representative flow cytometry histogram showing the MFI of GATA3 at 72 hours for *Cftr*^+/+^ and *Cftr*^−/−^ Th2 cells. (**E**) The quantified MFI of GATA3 in *Cftr*^+/+^ and *Cftr*^−/−^ Th2 cells at 72 hours (*n* = 5 mice per genotype). (**F**) Representative CD4^+^ populations showing the MFI of GATA3 at 72 hours in the presence of increasing doses of polarizing IL-4 (0–40 ng/mL) for *Cftr*^+/+^ and *Cftr*^−/−^ Th2 cells. (**G** and **H**) GATA3 MFI and secreted IL-13 from cellular supernatant in cultured *Cftr*^+/+^ (black) and *Cftr*^−/−^ (red) Th2 cells in the presence of increasing doses of IL-4 (0–40 ng/mL). (**C** and **E**) Data are shown as mean ± SD. Statistical analysis in **C** and **E** were performed using unpaired Student’s *t* test and, in **G** and **H**, by 4-parameter logistic regression algorithm (sigmoidal curve fit) to fit. For **G** and **H**, data are shown as mean values with the accompanying curve fit (solid line), the 95% CI displayed as a band as well as mean data points for each genotype and concentration. **P < 0.01 and ****P < 0.0001.

Given increased secreted Th2 cell cytokines and enrichment of IL-4 signaling–specific transcripts in *Cftr^−/−^* Th2 cells compared with *Cftr^+/+^* Th2 cells, we next tested the hypothesis that loss of CFTR results in augmented IL-4 signaling in CD4^+^ T cells. To test this hypothesis, we isolated naive CD4^+^ T cells from splenocytes of *Cftr^+/+^* and *Cftr^−/−^* mice, cultured the cells in Th2 polarizing conditions, and harvested the resulting Th2 cells at 72 hours for analytical flow cytometry (Figure 5A). Loss of CFTR in Th2 cells increased expression of the α subunit of the IL-4 transmembrane receptor (IL-4Rα; Figure 5, B and C) and upregulated expression of the downstream transcription factor target, GATA3 (Figure 5, D and E). Since IL-4 signaling induces GATA3 expression in CD4^+^ T cells (28), we next cultured naive CD4^+^ T cells with increasing doses of IL-4 (0–40 ng/mL) for 72 hours. *Cftr^−/−^* Th2 cells had a greater percentage of GATA3^+^CD4^+^ T cells at 72 hours at lower doses of IL-4 compared with *Cftr^+/+^* CD4^+^ T cells (Figure 5F). IL-4–induced GATA3 expression (Figure 5G; *P* < 0.0001) and secreted IL-13 in cellular supernatant (Figure 5H; *P* < 0.0001) was significantly increased in *Cftr^−/−^* CD4^+^ T cells compared with *Cftr^+/+^* CD4^+^ T cells via 4-parameter sigmoidal curve fit analyses, defined by a decreased EC_50_ (1.86 ng/mL [1.42–2.87] in *Cftr^−/−^* CD4^+^ T cells versus 5.316 ng/mL [3.40–10.86] in *Cftr^+/+^* CD4^+^ T cells for secreted IL-13 comparison). No differences in IL-4Rα expression were seen in *Cftr^+/+^* and *Cftr^−/−^* CD4^+^ T cells within the first 10 hours of activation (Supplemental Figure 9, A–G). These data demonstrate that CFTR negatively regulated GATA3 expression, in part through decreased IL-4 sensitivity of CD4^+^ T cells.

### Increased CFTR function decreased Th2-mediated allergic inflammation in a humanized CFTR mouse model.

**Figure 6:**
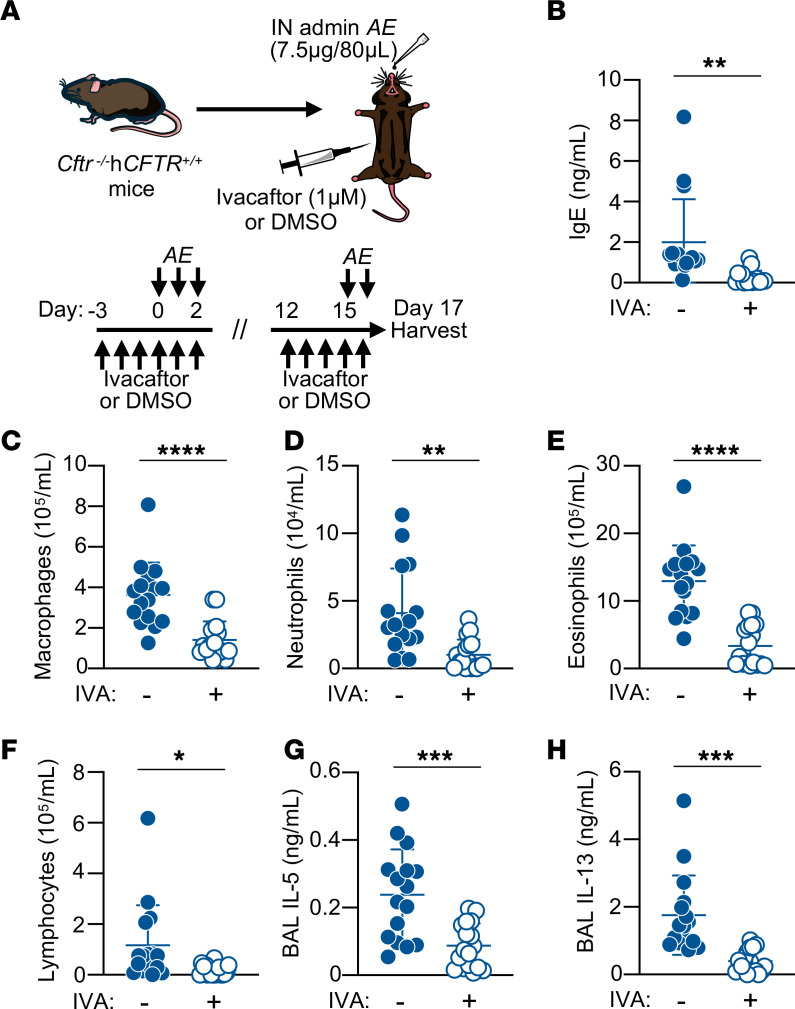
Increased CFTR function with ivacaftor decreases allergic inflammation in a humanized CFTR mouse model. (**A**) Schematic diagram showing an adaptive model of intranasal *AE*-induced inflammation and i.p. ivacaftor (IVA, 1 μM) or DMSO administration schedule in *Cftr*^−/−^*hCFTR^+/+^* mice. (**B**) IgE concentrations by ELISA in serum from sensitized/challenged mice treated with IVA (*n* = 17) or DMSO (*n* = 16). (**C**–**F**) The number of macrophages (**C**), neutrophils (**D**), eosinophils (**E**), and lymphocytes (**F**), in the BALF of *AE*-challenged mice treated with either IVA (*n* = 17) or DMSO (*n* = 16). (**G** and **H**) IL-5 and IL-13 by ELISA in BALF from *AE-*sensitized and challenged mice treated with either IVA (*n* = 17) or DMSO control (*n* = 16). Open circles represent IVA treated mice, and closed circles denote DMSO-treated control mice. Statistical analysis in **B**–**H** performed using unpaired Student’s *t* test. *P < 0.05, **P < 0.01, ***P < 0.001, and ****P < 0.0001.

Given our prior work demonstrating decreased human epithelial type 2 alarmin release with CFTR modulator treatment (15) and data demonstrating CFTR modulator–mediated decreased Th2 effector function in mouse *hCFTR* CD4^+^ T cells (Figure 2, G and H), we next hypothesized that treatment with the CFTR potentiator ivacaftor reduces allergic inflammation to *AE* in *hCFTR* mice. To test whether increased CFTR function can attenuate allergic inflammation, mice expressing either mouse *Cftr* or *hCFTR* were sensitized and challenged with *AE* (7.5 μg) using the adaptive model of allergic airway inflammation and given either once daily i.p. ivacaftor (10 mg/kg) or vehicle control (DMSO 5% v/v) leading up to and during sensitization and challenge (Figure 6A). Using IgE levels to broadly assess the adaptive immune response, we observed a significant decrease in serum total IgE amounts in ivacaftor-treated *hCFTR* mice compared with vehicle control–treated *hCFTR* mice (Figure 6B). The effect of ivacaftor treatment on *AE*-induced cellular inflammation was assessed in the BALF (Figure 6, C–F). BALF cell differential counts revealed significantly less recruitment of macrophages (Figure 6C), neutrophils (Figure 6D), eosinophils (Figure 6E), and lymphocytes (Figure 6F) in ivacaftor-treated compared with vehicle-treated *hCFTR* mice. No BALF cell type–specific percentage changes were noted in our comparison of the genotype or treatment groups, indicating the primary effect of ivacaftor was diminishing the magnitude of the type 2 response (Supplemental Figure 10, A–D). Consistent with these findings, ivacaftor treatment in *hCFTR* mice significantly decreased IL-5 (Figure 6G) and IL-13 (Figure 6H) cytokine secretion measured in BALF compared with vehicle control–treated mice. Importantly, no differences were noted between ivacaftor and vehicle-treated mice expressing mouse *Cftr* (*Cftr^+/+^*), demonstrating ivacaftor’s specificity for hCFTR (Supplemental Figure 11, A–G). These data demonstrate that in vivo ivacaftor treatment reduced type 2 airway inflammation to *AE*, requiring hCFTR expression to mediate the therapeutic effect.

### Increased CFTR function decreases GATA3 and IL-13 expression in human Th2 cells.

**Figure 7:**
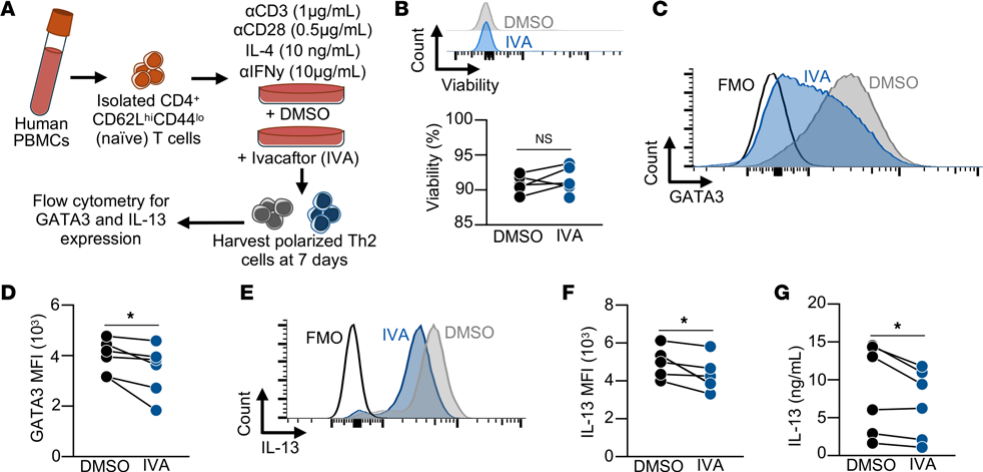
CFTR potentiation decreases Th2 GATA3 expression and IL-13 production. (**A**) Schematic diagram detailing the isolation and Th2 polarization of naive human CD4^+^ T cells used for flow cytometry and cytokine analysis. (**B**) Viability of ivacaftor (IVA) or DMSO (control) cultured human Th2 cells at 7 days. (**C**) Representative flow cytometry histogram showing the median fluorescence intensity (MFI) of GATA3 at 7 days for DMSO- and IVA-treated Th2 cells. (**D**) The quantified MFI of GATA3 in DMSO- and IVA-treated Th2 cells at 72 hours (*n* = 6 paired human samples). (**E**) Representative flow cytometry histogram showing the median fluorescence intensity (MFI) of IL-13 at 7 days for DMSO- and IVA-treated Th2 cells. (**F**) The quantified MFI of IL-13 in DMSO- and IVA-treated Th2 cells at 72 hours (*n* = 6 paired human samples). (**G**) IL-13 by ELISA in cellular supernatant from cultured DMSO- and IVA-treated Th2 cells. Statistical analysis in **B**, **D**, **F**, and **G** was performed using paired Student’s *t* test. *P < 0.05.

To assess whether the findings of decreased type 2 immunity with CFTR potentiation in the mouse model translate to T cell–mediated inflammation in humans, naive CD4^+^ T cells were isolated from healthy human PBMCs and cultured in Th2 polarizing conditions for 7 days in the presence of ivacaftor or DMSO control (Figure 7A). The percent of viable cells was similar at 7 days between DMSO- and ivacaftor-treated CD4^+^ T cells (Figure 7B). To test the hypothesis that CFTR potentiation decreases Th2 effector function, cultured cells were processed for analytical flow cytometry and secreted cytokine analysis (Supplemental Figure 12). CFTR potentiation with ivacaftor in Th2 cells led to significantly decreased expression of GATA3 (Figure 7, C and D) and intracellular IL-13 compared with DMSO control–treated cells (Figure 7, E and F). Consistent with these findings, secreted IL-13 measured in the cultured cellular supernatants was significantly decreased in ivacaftor-treated CD4^+^ T cells compared with DMSO-treated cells (Figure 7G). Taken together, these data demonstrate that CFTR modulation decreased Th2 effector function and suggest that targeting CFTR may represent an adjunct to current therapies in allergic disease.

## Discussion

Clinically, individuals with CF have increased rates of type 2 immune diseases including asthma, allergic rhinitis, and allergic bronchopulmonary aspergillosis (ABPA) (3, 29). Collectively these diseases represent hyperinflammatory responses defined by increased levels of IL-4, IL-13, and IgE (30–32). Prior studies have demonstrated a skewing of CF CD4^+^ T cells toward Th2 responses (8, 13, 15, 16, 33). However, the mechanisms pertaining to CFTR expression in CD4^+^ T cells and determinants for increased effector phenotypes are not well characterized. Here, we used mouse cellular and in vivo models deficient in CFTR, which lack any spontaneous airway infection, to study the effect of CFTR in Th2 effector function and the role of CD4^+^ T cell–specific CFTR in type 2 adaptive immunity. Our results show that CFTR expression is temporally associated with CD4^+^ T cell activation and that CFTR negatively regulates Th2 effector function, in part through negative regulation of the IL-4/GATA3 axis. These results are further supported by increased type 2 inflammation to *AE* sensitization and challenge in a mouse model of CD4^+^ T cell–specific loss of CFTR compared with CFTR-sufficient controls. Importantly, we report that pharmacologic potentiation of CFTR with the FDA-approved compound ivacaftor is associated with decreased *AE*-induced allergic inflammation in a humanized CFTR mouse model and decreased GATA3 and IL-13 expression in human CD4^+^ T cells.

Here we build on previous studies that have shown CFTR to be expressed in CD4^+^ T cells (34–36). Consistent with our findings of CFTR expression in an immortalized CD4^+^ lymphocyte cell line (Jurkat), early studies of CF demonstrated evidence of a functional cAMP-regulated chloride channel consistent with CFTR in the same cells (37). To our knowledge, these early studies in CD4^+^ T cells were the first to show nonepithelial cell CFTR expression and highlight the early recognition of CFTR intrinsic effects in CD4^+^ T cells. Despite early identification of functional CFTR expression in lymphocytes, our understanding of the contributions of CFTR to CD4^+^ Th2 effector function remain limited. CFTR has previously been proposed to mediate calcium flux across the T cell plasma membrane within seconds of TCR activation, thereby regulating calcium–sensitive gene expression pathways (10). In the present studies, no *Cftr* transcript or CFTR protein was observed in the naive T cell and was only present hours following TCR ligation. Therefore, the augmented calcium flux in CFTR-deficient T cells seen in the prior studies cannot be explained by our studies and likely reflect differences in the CF model. Importantly, the prior studies used mice harboring a misfolded ΔF508 mutation of CFTR, which is known to have residual functional activity yet is improperly retained in the endoplasmic reticulum and Golgi networks (38, 39), resulting in endoplasmic reticulum condensation and clustering of large-conductance cation channels. While disease relevant, these prior observations may reflect changes specific to improper CFTR localization and/or activity as opposed to the intrinsic effect of normal CFTR. Conserved in both the prior and current studies is augmented Th2 effector cytokine production in the setting of abnormal or absent CFTR (10, 13).

How might loss of CFTR contribute to the augmented Th2 response seen in our model? *Cftr^−/−^* CD4^+^ T cells have increased Th2 effector function compared with *Cftr^+/+^* controls. No detectable *Cftr* transcript or protein was noted in naive primary cell cultures, suggesting that the major effect of CFTR during polarization occurs hours after TCR stimulation following required transcription, translation, and trafficking of the CFTR protein. Despite having similar initial surface levels of the cytokine receptor for IL-4 (IL-4R), *Cftr^−/−^* CD4^+^ T cells demonstrated increased sensitivity to IL-4, with increased GATA3 expression compared with *Cftr^+/+^* CD4^+^ T cells by 72 hours. Taken together, we hypothesize that CFTR could be a target of GATA3 transcription factor activity, which then acts as a feedback inhibitor against stable Th2 commitment. Indeed, within the CFTR promoter exists a putative GATA3 binding motif at site –607 bp respective to the CFTR transcription start site. Further studies will be required to integrate whether GATA3 has transcriptional activity on the *Cftr* promoter. CFTR expression may subsequently modify IL-4R signaling during polarization through direct ion dysregulation via chloride and/or bicarbonate movement, via CFTR’s direct protein-protein interactions with downstream signaling proteins important for IL-4 signaling such as PTEN-PI3K, or via intracellular fluid homeostasis. More investigations are needed to clarify the exact mechanism of CFTR’s regulation of Th2 commitment and whether there is an effect of CFTR mutational class on Th2 effector function. Together, the prior and current studies suggest that multiple mechanisms of CD4^+^ T cell dysfunction may exist in CF disease, including contributions from the lack of CFTR and localization of CFTR.

A CFTR/IL-4R signaling axis has been implicated in other cell types responsive to IL-4. Recent studies have noted a hyperresponsiveness to IL-4 in CFTR-deficient compared with CFTR-sufficient B cells (8), with increased expression of BAFF, CXCR4, and IL-6 in IL-4–treated *Cftr^−/−^* B cells compared with *Cftr^+/+^* B cells (40). Equally interesting is the observation that IL-4 can induce *CFTR* expression and upregulate cAMP-dependent current in human epithelial models (41). Taken together, these observations support our hypothesis that IL-4R signaling may induce a possible CFTR-mediated “Th2 braking mechanism” while simultaneously driving canonical Th2 effector function. Further studies aimed at understanding this potential “rheostat” mechanism involving CFTR function and IL-4/GATA3 regulation will be important.

The ability of CFTR to suppress Th2 responses arising from allergy, both through epithelial-derived cytokines and Th2 cell function, provides a unique opportunity to therapeutically target a novel multicellular immunomodulatory pathway. Recently, CFTR modulators designed to improve channel function in persons with different CFTR mutations has revolutionized the clinical treatment of CF (42). CFTR potentiators such as ivacaftor increase the open probability of CFTR channels with a gating mutation, and can also increase WT CFTR function (43, 44). We show that the CFTR potentiator ivacaftor in a mouse model of allergic disease significantly reduced allergic inflammation through reductions in recruited eosinophils and lymphocytes, as well as through production of IgE compared with vehicle treated mice. Together with our prior findings demonstrating that CFTR modulation reduces epithelial release of the potent type 2 cytokine IL-33 and our present studies showing significant GATA3 and IL-13 reductions in human Th2 cells treated with ivacaftor compared with DMSO controls, CFTR modulation has the potential to broadly target allergic disease. Indeed, we and others have shown that individuals with CF have significant reductions in type 2 biomarkers after initiation of CFTR modulator therapy (3, 45). Whether these reductions in type 2 biomarkers could extend to a non-CF population and whether CFTR function can modify Th2 effector function in previously committed Th2 cells remains unstudied. CFTR-targeting compounds may serve as potential adjuvants to current allergy therapies by mechanistically targeting nonredundant pathways in allergic disease.

Our studies have both advantages and limitations. Advantages include the following: (a) We used comparative methodologies highlighting a conserved effect of CFTR on Th2 effector function in both humans and mice. (b) Cre recombinase–driven deletion of T cell–specific CFTR in mice provided a powerful approach to assess CD4^+^ T cell CFTR function in allergy, a method that would be more challenging in larger-animal CF models. (c) We took advantage of the lack of spontaneous lung disease in mice to determine the primary effect of allergen challenge on Th2-driven inflammation. (d) Using a recently developed humanized CFTR mouse model, we demonstrated an in vivo ivacaftor-mediated decrease in allergy through reductions in type 2 cytokines (IL-5 and IL-13) and total serum IgE. Limitations include the following: (a) We cannot exclude the possibility of CFTR effects on other cell types of the innate and adaptive immune system in our allergy model. CFTR expression and function has been implicated in other relevant inflammatory cell types including macrophages, B cells, and neutrophils (40, 46–50). We utilized *AE* as a potent stimulus of eosinophilic and lymphocytic inflammation, but other common aeroallergens such as host dust might may have differential effects on airway allergy in CF based on the resultant cellular inflammation. The ability of CFTR targeting therapies to alter heterogenous cell populations may be advantageous, but future studies will be required to understand how CFTR modulating therapy affects other types of inflammation. (b) We did not assess a role for other T cell subsets including Th1 and Th17 cells in this model. Although we focused primarily on the direct effects of Th2-mediated inflammation, previous studies demonstrated a Treg functional deficiency in both CF human and *Cftr*^−/−^ mouse models (51), and our time course analysis of CFTR expression did reveal a significant but, relative to Th2 cells, small induction of *Cftr* mRNA in Tregs. It may be possible that T cell differentiation is broadly altered by CFTR loss, and further studies will be required to understand CFTR contributions to the differentiation and effector function of other T cell subsets. (c) We performed in vitro polarization of naive CD4^+^ cells to limit non–CD4^+^ T cell contributions to the polarized Th2 cell; however, we cannot exclude that naive CD4^+^ T cells may be inherently reprogrammed, making them more sensitive to Th2 polarizing conditions. Indeed, prior studies have found that TCR ligation alone in naive CF CD4^+^ T cells results in increased Th2 skewing compared with non-CF controls (13). Assessing the acute changes between CD4^+^ T cell naivety and activation serve as attractive areas of further study to better understand not only the contributions of CFTR to Th2 effector identity but also whether CFTR targeting therapies are most viable as a preventative or relief therapy in allergic disease.

In summary, our findings demonstrate that CD4^+^ T cells expressed CFTR following stimulation and CD4^+^ T cell loss of CFTR significantly increased Th2 effector function in vitro and augmented in vivo allergic inflammation to *AE*. Furthermore, we demonstrated that CFTR functions as a negative regulator of Th2 effector function, in part, by reducing the IL-4/GATA3 signaling axis. The recently approved CFTR potentiator, ivacaftor, decreased inflammation in an in vivo mouse allergy model and reduced GATA3 expression and effector function in in vitro polarized human Th2 cells. The CFTR modulator drug class may provide pharmacological approaches to managing common and persistent type 2 inflammation in CF via epithelial and CD4^+^ T cell mechanisms, while more broadly representing a new class of therapies to be repurposed for allergic disease.

## Methods

### Sex as a biological variable.

Our study examined the effect of CFTR deficiency in female mice because female animals exhibited heightened type 2 inflammation and less variability in phenotype compared with male mice. Our CFTR potentiation studies in humanized CFTR mice and human T cells examined both male and female animals/humans, and similar findings are reported for both sexes.

### Study design.

The objective of this study was (a) to determine the role of CFTR in Th2 cell function and Th2 cell–mediated allergic inflammation, (b) elucidate the underlying molecular Th2 cell signaling mechanisms, and (c) test the therapeutic potential of a clinically approved CFTR potentiator in allergic disease. Experimental approaches comprise in vitro cellular studies in human and mouse CD4^+^ T cells, next-generation bulk RNA-Seq of mouse Th2 cells, and in vivo allergen studies in mice. Two lines of in vivo experimentation designed to elicit adaptive type 2 inflammation were performed: (a) PBS- or *AE*-challenged *CD4^Cre+^Cftr^fl/fl^* or *CD4^Cre−^Cftr^fl/fl^* mice and (b) *AE*-challenged mouse *Cftr* or *hCFTR* expressing mice treated with vehicle control or the CFTR potentiator ivacaftor. For in vitro experimentation, naive *Cftr^+/+^* and *Cftr^−/−^* mouse CD4^+^ T cells and naive *hCFTR* and *hCFTR^ΔF508^* mouse CD4^+^ T cells were isolated from mouse splenocytes and cultured in Th2 polarizing conditions, a subset of which were treated with pharmacological inhibitors of activators of CFTR. Human naive CD4^+^ T cells were isolated from PBMCs, cultured in Th2 polarizing conditions, and treated with either DMSO control or ivacaftor. For RNA-Seq studies, in vitro polarized *Cftr^+/+^* and *Cftr^−/−^* Th2 cells were submitted for analysis.

### Animals.

For in vitro studies, *Cftr^tm1^ ^Unc^*Tg(FABPCFTR)1Jaw/J mice were obtained from The Jackson Laboratory (stock no. 002364). These mice are KOs for the mouse *Cftr* gene (*Cftr*^−/−^) but express hCFTR in the gut under control of the fatty acid binding protein 1 (FABP1) promoter, which prevents acute intestinal obstruction. For in vivo studies requiring T cell–specific deletion of CFTR, adult female *CD4^Cre+^Cftr^fl/fl^* or their corresponding *CD4^Cre−^Cftr^fl/fl^* littermates were used for experiments. For in vivo CFTR potentiation studies, adult male and female mice deficient in mouse CFTR (*Cftr^−/−^*) but possessing a *hCFTR* transgene (*hCFTR*) or same-strain (C57BL/6J) housed *Cftr^+/+^* controls lacking the *hCFTR* transgene were used. Additionally, mice possessing a mutation in the *hCFTR* gene resulting in a deletion of phenylalanine 508 (*hCFTR^ΔF508^*) and *hCFTR* mice were used for in vitro experiments (17, 18). All animals were maintained under specific pathogen–free conditions and 12-hour light/dark cycles with free access to food and water. The number of experimental replicates is indicated in the figure legends.

### CD4^+^ T cell culture.

CD4^+^ T cells were purified from the splenic cells of *Cftr^+/+^* and *Cftr^−/−^* littermate mice by either a mouse pan-CD4^+^ T cell isolation kit (19852, Stemcell Technologies) or mouse naive CD4^+^ T cell isolation kit (19765, Stemcell Technologies; Supplemental Figure 2). The purified pan-CD4^+^ or CD62L^hi^CD44^lo^CD4^+^ T cells were resuspended at 1 × 10^6^ cells/mL in RPMI 1640 medium (Mediatech) supplemented with 10% FBS (HyClone), 4 mM of L-glutamine (Thermo Fisher Scientific), 1 mM of sodium pyruvate (Thermo Fisher Scientific), 55 μM of β-mercaptoethanol (2-ME; Thermo Fisher Scientific), 10 mM of HEPES (Thermo Fisher Scientific), 100 U/mL penicillin (Thermo Fisher Scientific), and 100 μg/mL streptomycin. The cells were stimulated with plate-bound anti-CD3 (1 μg/mL; 145-2C11, BD Biosciences) and anti-CD28 (1 μg/mL; 37.51, BD Biosciences) in 24-well flat-bottom plates for initial TCR ligation studies. For polarization and time course studies, naive T cells were provided stimulation as follows: Th2, anti–IFN-γ (10 μg/mL; XMG1.2, BD Biosciences) and mouse IL-4 (10 ng/mL; Stemcell Technologies); Th1, anti–IL-4 (10 μg/mL; 11B11, BD Biosciences), and mouse IL-12 (10 ng/mL; R&D Systems); and Th17, human TGF-β (0.5 ng/mL; Stemcell Technologies), mouse IL-23 (10 ng/mL; Invitrogen), mouse IL-6 (40 ng/mL; Stemcell Technologies), mouse IL-1b (10 ng/mL; Stemcell Technologies), anti–IL-4 (10 μg/mL; 11B11, BD Biosciences), and anti–IFN-γ (10 μg/mL; XMG1.2, BD Biosciences). Tregs were polarized and restimulated with anti-CD3 (1 μg/ mL), human IL-2 (100 IU/mL), and recombinant human TGF-β (0.5 ng/mL). For time course studies, cells were harvested for cellular RNA at 0, 6, 18, and 72 hours after polarization as well as 6 hours after restimulation (78 hours) with TCR ligation and subset-specific stimulation as above. For Th2-specific studies, after 3 days, the culture supernatant was harvested for multiple cytokine ELISAs. Cellular RNA was collected for RNA-Seq as described below. For humanized CFTR CD4^+^ T cell studies, cells from *hCFTR^ΔF508^* and *hCFTR* mice were isolated and polarized in Th2 conditions as above, in the presence of the CFTR potentiator ivacaftor (1 μM; Selleck Chemicals), and the CFTR correctors elexacaftor (3 μM; Selleck Chemicals) and tezacaftor (3 μM; Selleck Chemicals, elexacaftor-tezacaftor-ivacaftor [ETI]) or DMSO control (%5 v/v).

For human cell CD4^+^ T cell studies, human PBMCs were prepared from whole blood of healthy donors by Ficoll density gradient centrifugation (800*g*) using Lymphoprep density gradient medium (StemCell Technologies) and SepMate-50 tubes (StemCell Technologies). For the purification of human naive CD4^+^ T cells, an EasySep human naive CD4^+^ T cell isolation kit II (StemCell Technologies) was used. The purification procedures were carried out according to the supplier’s instructions. For expansion, cells were cultivated in ImmunoCult-XF T Cell Expansion Medium (StemCell Technologies). The media were supplemented with ImmunoCult Human Th2 Differentiation Supplement (containing recombinant human IL-4 and mouse anti–human IFN-γ, StemCell Technologies) and 100 IU/mL IL-2 (NIH). T cells were activated by 25 μL/mL ImmunoCult Human CD3/CD28/CD2 T Cell Activator reagent at a cell density of 1 × 10^6^ cells/mL (Stemcell Technologies). After 7 days, cells were collected for analytical flow cytometry and the culture supernatant was harvested for multiple cytokine ELISAs.

### RNA-Seq.

Cellular RNA was isolated from cultured mouse *Cftr*^+/+^ and *Cftr^−/−^* Th2 cells representing 3 biologic replicates for each genotype. Genomic DNA was digested using DNase I (Qiagen). RNA samples were then quantified using fluorimetry (Qubit 2.0 fluorometer; Invitrogen), and RNA quality was assessed using an Agilent BioAnalyzer 2100 (Agilent Technologies). Only samples with RNA integrity numbers > 8.0 were used. Library preparation and sequencing were conducted in the Vanderbilt Technologies for Advanced Genomics (VANTAGE) core facility. Briefly, an Illumina Ribo-Zero Plus rRNA Depletion kit was used to isolate rRNA-depleted RNA. The samples were then reverse transcribed to create cDNA. The cDNA was fragmented, blunt-ended, and ligated to indexed adaptors. Following quantification of the cDNA generated for the library, the samples were clustered and loaded equally over 2 lanes on an Illumina NovaSeq6000 Sequencing system (Illumina Inc.), which generated on average > 45 million paired reads of 150 bp. For analysis, raw data were aligned and counted to the GRCm38 reference genome by the Dragen workflow provided by VANTAGE. For downstream analysis, all samples were normalized by trimmed mean of M-values (TMM) with DESeq2 package. PCA plot was generated from these normalized values, log_2_ fold change values were calculated, and its output was used for volcano plot construction and gene set enrichment for KEGG pathway and Harmonizome gene set analysis (Ma’ayan Laboratory; Icahn School of Medicine at Mount Sinai, New York, New York, USA). Significant differential expression was determined in genes with FDR-adjusted *P* < 0.01 and fold change ± 1.5. Additional GSEA was performed with the clusterProfiler R package (52) on a ranked list of differentially expressed genes sorted by log_2_(fold change) using GO:BP and KEGG curated gene sets. Gene sets with an adjusted *P* < 0.05 were considered significant. The Gene-Concept Network was generated from highly enriched KEGG gene sets of interest relating to cell signaling (KEGG_ASTHMA, KEGG_JAK_STAT_SIGNALING_PATHWAY, and KEGG_CYTOKINE_CYTOKINE_RECEPTOR_INTERACTION). Hierarchical clustering of the 30 most enriched GO:BP terms by Jaccard’s similarity index was performed with clusterProfiler, DOSE, and enrichplot R packages. Terms were grouped into 4 clusters with 1 label term per cluster. Both plots were visualized with ggplot2.

### Alternaria-extract challenge in mice.

Mice were anesthetized under continuous delivery of isoflurane and oxygen into a chamber placed within a sterile hood. For the adaptive model, from day 0 to day 2, either 7.5 μg (protein amount) of *AE* (Stallergenes Greer) in 80 μL of PBS or 80 μL of PBS as vehicle were administered intranasally to anesthetized mice. *AE* (7.5 μg protein amount) in 80 μL of PBS or 80 μL of PBS intranasal challenge occurred on days 15 and 16. For ivacaftor treatment experiments, *AE*-challenged mice from days –3 to 2 and days 12 to 16 received either once-daily ivacaftor (10 mg/kg; Selleck Chemicals) or vehicle control (5% DMSO v/v) intraperitoneally. Whole lungs and BALF were harvested on day 17, 24 hours after the last challenge.

### Cytokine and IgE ELISAs.

Murine IL-5, murine IL-13, and murine IgE were assayed using Quantikine ELISA kits following manufacturer’s instructions (R&D Systems).

### Flow cytometry.

In vitro polarized mouse *Cftr*^+/+^ and *Cftr^−/−^* Th2 cells (day 3 of culture) and human Th2 cells (day 7 of culture) were incubated in the presence of 1 μL GolgiPlug (BD Biosciences) for 4 hours. Cells were then resuspended in a buffer containing 2% FBS, and 2 mM EDTA (Invitrogen) in PBS before being incubated with fixable Aqua dead cell stain (Invitrogen, L34957) before staining with fluorophore-conjugated antibodies. Foxp3/Transcription factor Staining kit (00-5523-00, Thermo Fisher Scientific) was used to stain GATA3 and other intracellular proteins according to the manufacturing protocol. Murine cells were stained with antibodies against the following molecules (clone, conjugate; source): CD4 (GK1.5, BUV395, BD Biosciences), CD3 (145-2C11, APC-Cy7, BD Biosciences), CD45 (30-F11, BB700, BD Biosciences), CD124 (Astra-1, AF488, Invitrogen), IL-4 (11B11, BV650, BD Biosciences), IL-13 (eBio13A, PerCP-ef710, Invitrogen), and GATA3 (TWAJ, eFlour 660, Invitrogen). Human cells were stained with antibodies against the following molecules: CD4 (RPA-T4, Super Bright 780, Invitrogen), GATA3 (TWAJ, eFlour 660, Invitrogen), and IL-13 (85BRD, Alexa Flour 647, Invitrogen). Data were acquired on a BD LSRFortessa SORP flow cytometer (Becton Dickinson) or Cytek Aura spectral flow cytometer (Cytek). Flow data were analyzed using FlowJo software (Tree Star Inc.).

### Statistics.

All data were analyzed with GraphPad Prism 9 (GraphPad Software). Data are expressed as individual data points ± SD. Unless otherwise noted, for analyses that compared 2 groups, we used an unpaired 2-tailed Student’s *t* test. Statistical significance for more than 2 genotypes and more than 2 conditions (*AE* versus phosphate-buffered saline [PBS] or CFTR modulators versus vehicle control) was assessed by 2-way ANOVA with Bonferroni multiple-comparison test. Values of *P* < 0.05 were considered significant between 2 groups. For RNA-Seq analysis, gene counts were normalized using TMM, and significant differential expression was determined in genes with FDR-adjusted *P* < 0.01 and fold change ± 1.5.

### Study approval.

All animal use procedures were approved by the IACUC of Vanderbilt University Medical Center. Animals were randomized to different treatment groups. Sample sizes were chosen empirically based on statistical power calculations. Investigators performing the animal experiments were not blinded to group information. Human PBMCs were obtained from healthy individuals with no prior history of allergy or use of allergy targeting medications after written informed consent and approval by The Vanderbilt University IRB.

### Data availability.

FASTQ files containing the raw RNA-Seq reads were deposited in the National Center for Biotechnology Information (NCBI) Sequence Reads Archive (SRX2630649[2-7]), with an accompanying BioProject ID (PRJNA1169855). All other data associated with this study are present in the paper or the Supplemental Methods, with data for all data points shown in graphs included in the associated Supporting Data Values file. For additional information, see Supplemental Methods.

## Author contributions

Conceptualization was contributed by MR, DPC, AEN, CAH, DCN, MHK, and RSP. Methodology was contributed by MR, DPC, CAH, DCN, MHK, and RSP. Investigation was contributed by DPC, CMT, MR, JZ, ST, WZ, MA, and DMY. Visualization was contributed by DPC, CMT, and MR. Funding acquisition was contributed by DPC and RSP. Project administration was contributed by JZ and DMY. Supervision was contributed by DPC and RSP. Writing of the original draft was contributed by MR, DPC, DCN, MHK, and RSP. Review and editing of the manuscript were contributed by DPC, CMT, MR, JZ, ST, WZ, MA, DMY, AEN, CAH, DCN, MHK, and RSP.

## Supplementary Material

Supplemental data

Unedited blot and gel images

Supporting data values

## Version 1 03/25/2025

In-Press Preview

## Version 2 05/08/2025

Electronic publication
