# Supplementary material for: CFTR negatively reprograms Th2 cell responses, and CFTR potentiation restrains allergic airway inflammation
Source: JCI Insight. 2025 Mar 25;10(9):e191098. doi: 10.1172/jci.insight.191098 (PMC12128969; doi:10.1172/jci.insight.191098)
Supplement: Supplemental data [file jciinsight-10-191098-s257.pdf]

Supplementary Materials for

CFTR negatively reprograms Th2 cell responses and CFTR potentiation restrains allergic airway inflammation

Mark Rusznak, Christopher M. Thomas, Jian Zhang, Shinji Toki, Weisong Zhou, Masako Abney, Danielle M. Yanda, Allison E. Norlander, Craig A. Hodges, Dawn C. Newcomb, Mark H. Kaplan, R. Stokes Peebles, Jr., Daniel P. Cook

Corresponding author: Daniel P. Cook, daniel-p-cook@uiowa.edu

The PDF file includes:

Materials and Methods
Figs. S1 to S12
Tables S1

Other Supplementary Material for this manuscript includes the following:

Supporting Data Values file

Materials and Methods

qPCR

RNA from *Cftr*$^{+/+}$ and *Cftr*$^{-/-}$ cultured CD4^{+} T cells were harvested in RLT lysis buffer from the RNeasy Mini Kit (Qiagen #74104). The lysis solution was purified by spinning the sample through QIAshredder columns (Qiagen #76956) and then purifying the RNA using RNeasy Mini Kit. Concentration of RNA was measured using a ThermoFisher Nanodrop. Complementary DNA (cDNA) was synthesized from RNA using SuperScript IV VILO Master Mix (Catalog # 11756050). A ThermoFisher nanodrop was used to quantify the amount of cDNA synthesized. Expression of mouse *Cftr* was analyzed using TaqMan primers (Mm01156903_m1) spanning exon 10 and 11 and normalized to *Gapdh* expression. Normalized fold expression was calculated using ΔΔCT method. For gel visualization, the PCR products were then separated according to size on a 1.5% agarose gel and visualized using SYBR Safe DNA Stain (EDVOTEK) against a 100 bp ladder (Life Technologies) over an ultraviolet light source.

CFTR immunoprecipitation and immunoblot

Cultured Jurkat cells were lysed with 300 µl of RIPA buffer containing phosphatase and protease inhibitor. The lysates were then sonicated and centrifuged at 13,000 rpm, 4°C for 15 min, supernatant total protein was measured (Bio-Rad, Hercules, CA) and then frozen at −80°C until used. For immunoprecipitation, 50 µl of Dynabeads Protein G beads (Thermofisher) was incubated with 5 µg CFTR UNC450 antibody or 5µg of IgG1 isotype control. Beads were placed on magnet and washed. 20 mg of Jurkat lysate was added to both UNC450 and isotype control antibody groups. Beads were suspended on magnet and washed. The resulting Bead/Ab/Ag complexes were resuspended in SDS sample buffer and loaded into the wells of a 10% precast Mini-PROTEAN TGX gel (4561034, BIORAD, Hercules, CA). Proteins were transferred to nitrocellulose membrane using iBlot system (Thermo Fischer Scientific). After protein transfer, membranes were blocked with Odyssey TBS blocking solution (LI-COR Biosciences) for 1 hr at RT and probed with another mouse anti-CFTR monoclonal antibody (1:1,000, UNC596) As secondary, infrared labeled and donkey anti-mouse antibody were used (1:10,000, LI-COR). The antibody signal was imaged and quantified by fluorescence detection on a LI-COR Odyssey CLx imaging system.

Cell Immunofluorescence

Cftr$^{+/+}$ and *Cftr*$^{-/-}$ cultured CD4^{+} T cells were rinsed in PBS, cytospun onto slides, labeled with CellMask Orange plasma membrane stain (Invitrogen), and fixed with 4% paraformaldehyde in PBS. Samples were permeabilized with 0.1% Triton X-100 in PBS and again rinsed with PBS. After fixation, non-specific binding sites were blocked with StartingBlock Blocking Buffer (ThermoFisher). Cells were stained with the anti-CFTR 596 primary antibody (1:100, UNC) and followed with goat anti-mouse Alexa Flour 488-conjugated secondary antibody (1:1,000, Invitrogen) to detect CFTR. Nucleic acids were stained with 4',6-diamidino-2-phenylindole (DAPI) (Molecular Probes). Preparations were mounted in an anti-fade solution Vectashield Mounting Medium (Vector Laboratories). Fluorescence was detected using confocal laser

scanning microscopy on an Olympus FV1000. Paired images were immunostained, visualized, and normalized to *Cftr*$^{-/-}$ controls. Images were processed using Image J software (NIH, Bethesda, MD).

Assessment of BALF inflammatory cells

BALF analysis was performed in anesthetized mice by instilling 800 µl of bacteriostatic normal saline through tracheotomy tube and withdrawing the fluid by syringe suction. Total cell counts were determined with a hemocytometer. Aliquots were cytospun and stained with Diff-Quick (American Scientific Products, McGaw Park, IL). Differential counts were performed by investigator blinded to animal group assignments, with morphologic criteria to classify the cells as eosinophils, lymphocytes, neutrophils, and mononuclear leukocytes.

Figure S1. Original images details

PCR products were analyzed on 2% Agarose gel, stained with SYBR Green nucleic acid dye (Invitrogen) and visualized under UV light. Western blot mages were obtained using the LI-COR Odyssey CLx imaging system. Headings above the figures correspond to the figure panels these blots were used for. Images represent the actual signal and used for the manuscript figures.

Full Gel for Figure 1A

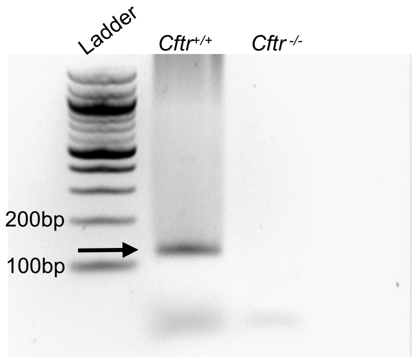

Fig S1.1. Full agarose gel electrophoresis of *Cftr* reverse-transcriptase PCR products from *Cftr*$^{+/+}$ and *Cftr*$^{-/-}$ CD4^{+} T cells.

Full blot for Figure 1D

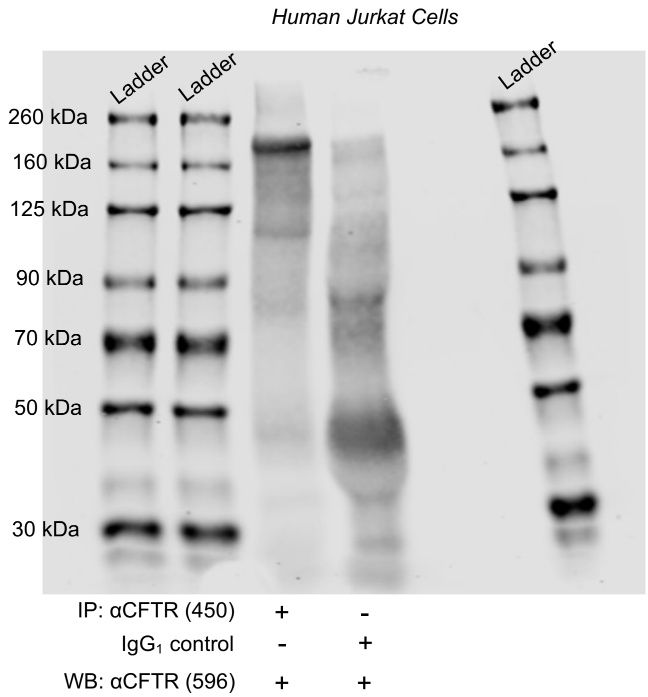

Fig. S1.2. The full image of western blot with markered IP conditions and ladders corresponding to Figure 1D.

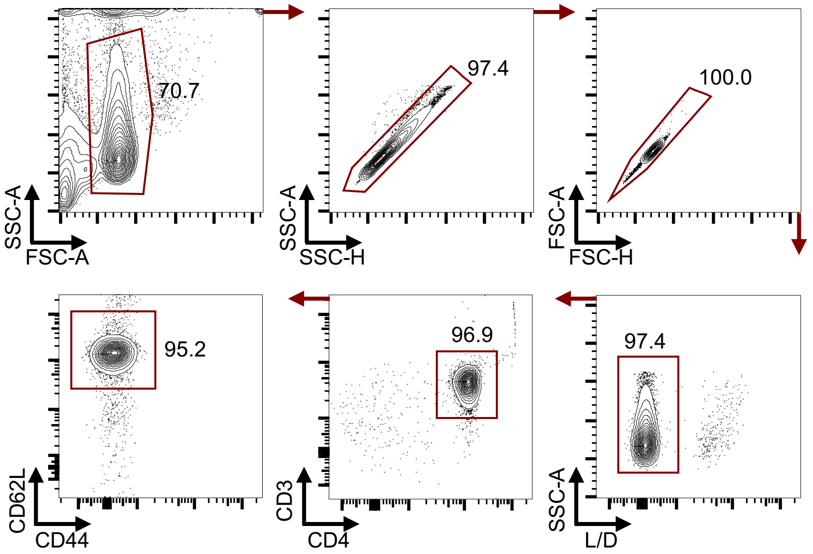

Fig. S2. Purity of splenocyte derived naïve CD4$^+$ T cell isolation protocol. Representative gating strategy and dot plot of magnetic bead enriched mouse CD62L^{hi}CD44^{lo}CD4$^+$ T cells, indicating > 95% purity naïve T cells from total CD4$^+$ T cell fraction. LIVE/DEAD Dead Cell Stain (L/D).

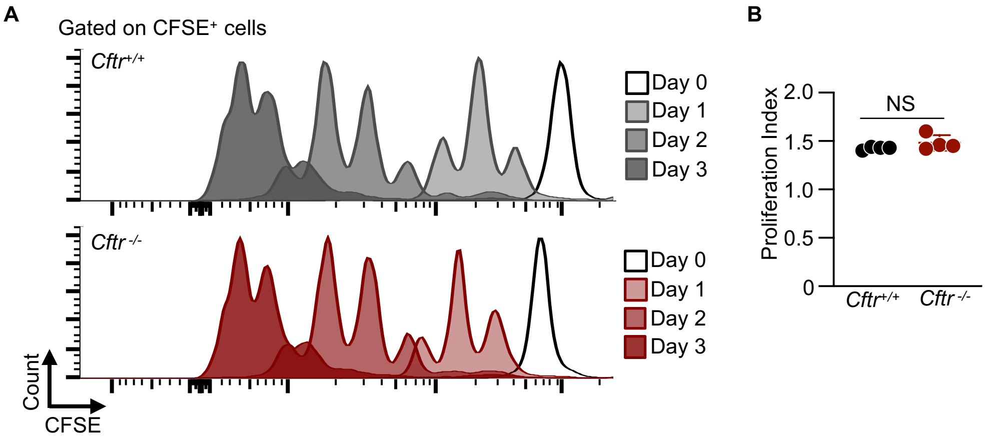

Fig. S3. Proliferation analysis of *Cftr*$^{+/+}$ vs. *Cftr*$^{-/-}$ CD4^{+} T cells in Th2 polarizing conditions. **(A)** Representative histograms of Carboxyfluorescein succinimidyl ester (CFSE) fluorescence in *Cftr*$^{-/-}$ (red) and *Cftr*$^{+/+}$ (grey) CD4^{+} T cells at day 0 (white), day 1 (light), day 2 (medium), and day 3 (dark) following TCR ligation and Th2 polarization. **(B)** Proliferative index in *Cftr*$^{-/-}$ (red) and *Cftr*$^{+/+}$ (grey) CD4^{+} T cells at day 3 ($n = 4$ mice per genotype). The proliferation index was calculated as the average number of divisions that those cells that divided underwent using Flowjo software. Statistical analysis (B) performed using unpaired student's t test, data plotted as mean ± SD. NS = not significant.

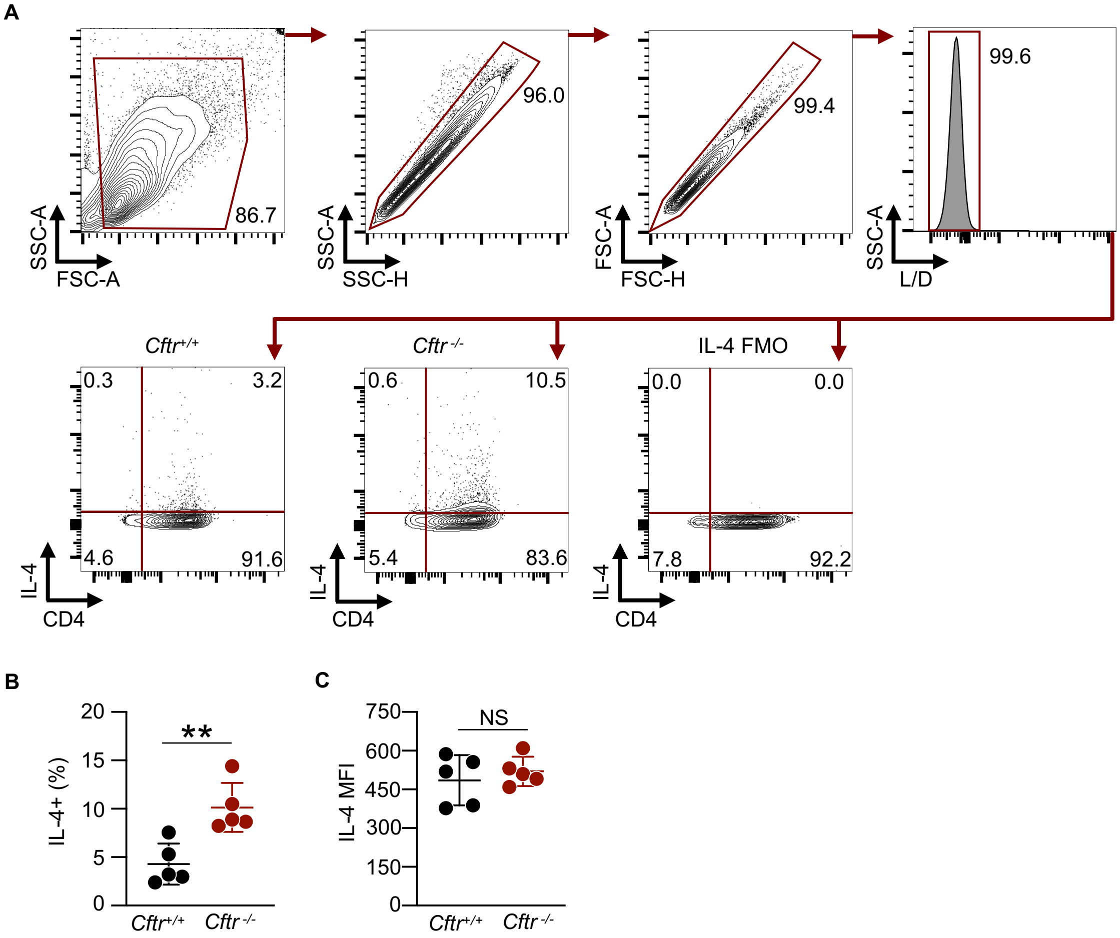

Fig. S4. Gating strategy and measurement of intracellular IL-4 in *Cftr*$^{+/+}$ vs. *Cftr*$^{-/-}$ CD4^{+} Th2 cells. (A) Representative gating strategy and dot plot for IL-4 intracellular cytokine and flow cytometry analysis of magnetic bead enriched human CD62L^{hi}CD44^{lo}CD4^{+} T cells cultured in Th2 polarizing conditions. **(B)** The percent IL-4 positive of total cells and **(C)** quantified MFI of IL-4 of IL-4 expressing cells in *Cftr*$^{+/+}$ and *Cftr*$^{-/-}$ Th2 cells at 72 hours ($n = 5$ mice per genotype). LIVE/DEAD Dead Cell Stain (L/D). Statistical analysis (B-C) performed using unpaired student's t test, data plotted as mean ± SD. $^{**}P < 0.01$, and NS = not significant.

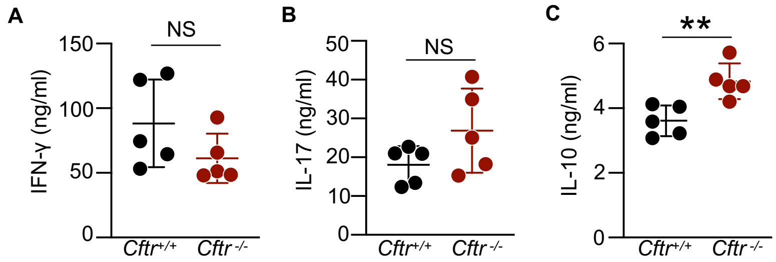

Fig. S5. Hallmark cytokine secretion in *Cftr*$^{+/+}$ vs. *Cftr*$^{-/-}$ CD4^{+} Th1, Th17, and Treg polarized CD4^{+} T cells. (A) IFN-γ, **(B)** IL-17, and **(C)** IL-10 by ELISA in cellular supernatant from *Cftr*$^{+/+}$ and *Cftr*$^{-/-}$ CD4^{+} T cells grown in culture stimulated with Th1, Th17, and Treg polarizing conditions, respectively ($n = 5$ mice per genotype). Data plotted as mean ± SD. Statistical analysis performed using unpaired student's t test. **$P < 0.01$, and NS = not significant.

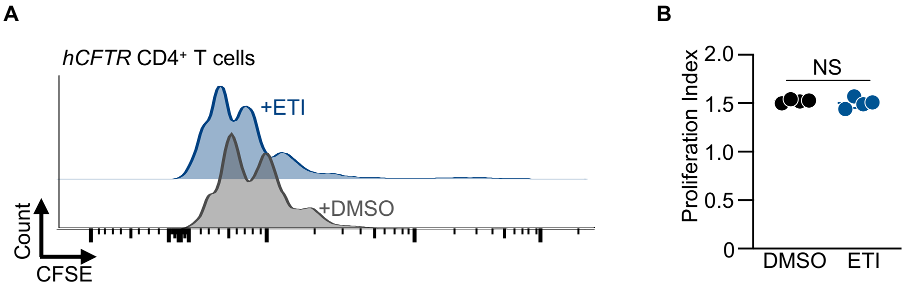

Fig. S6. Proliferation analysis of CFTR modulator treated vs. DMSO *hCFTR* CD4$^+$ T cells in Th2 polarizing conditions. (A) Representative histograms of Carboxyfluorescein succinimidyl ester (CFSE) fluorescence in CFTR correctors elexacaftor (3 µM) and tezacaftor (3 µM, elexacaftor-tezacaftor-ivacaftor [ETI]) treated (blue) and DMSO treated (grey) *hCFTR* CD4$^+$ T cells at day 3 following TCR ligation and Th2 polarization. **(B)** Proliferative index in ETI (blue) and DMSO (grey) CD4$^+$ T cells at day 3 ($n = 4$ mice per genotype). The proliferation index was calculated as the average number of divisions that those cells that divided underwent using Flowjo software. Statistical analysis (B) performed using unpaired student's t test, data plotted as mean ± SD. NS = not significant.

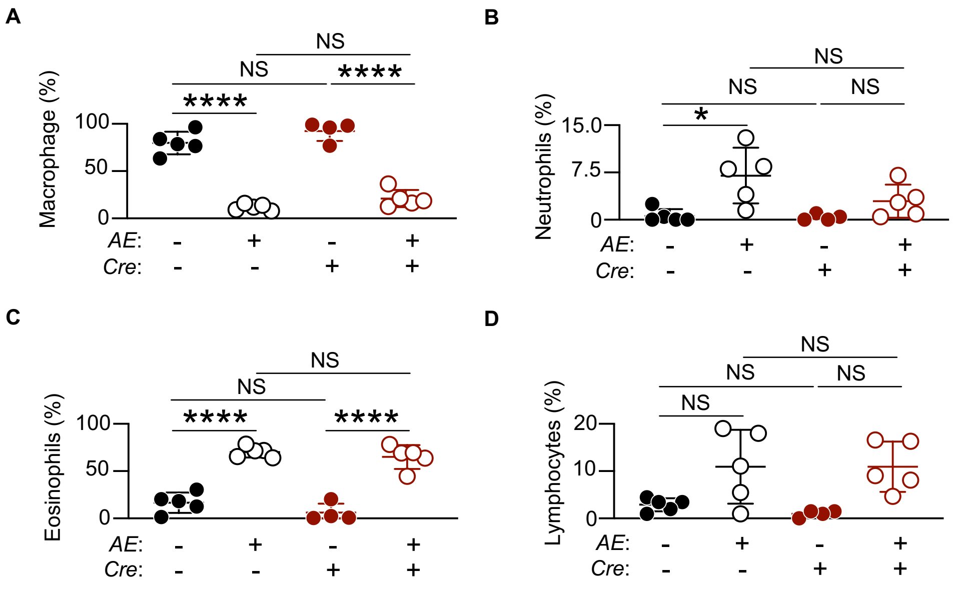

Fig. S7. Immune cell abundance in BAL from T cell specific CFTR deficient mouse experiments. The percentages of **(A)** macrophages, **(B)** neutrophils, **(C)** eosinophils, and **(D)** lymphocytes, in the BALF of phosphate-buffered saline (PBS) or *AE*-challenged $CD4^{Cre-}Cftr^{fl/fl}$ and $CD4^{Cre+}Cftr^{fl/fl}$ mice ($n = 4$–5 per genotype per condition). Open circles represent *AE* sensitized and challenged mice and closed circles denote PBS control mice. Statistical analysis in (A-D) by one-way ANOVA followed by Tukey's honestly significant difference (HSD) post hoc test for multiple comparisons. $^{*}P < 0.05$ and $^{****}P < 0.0001$. NS = not significant.

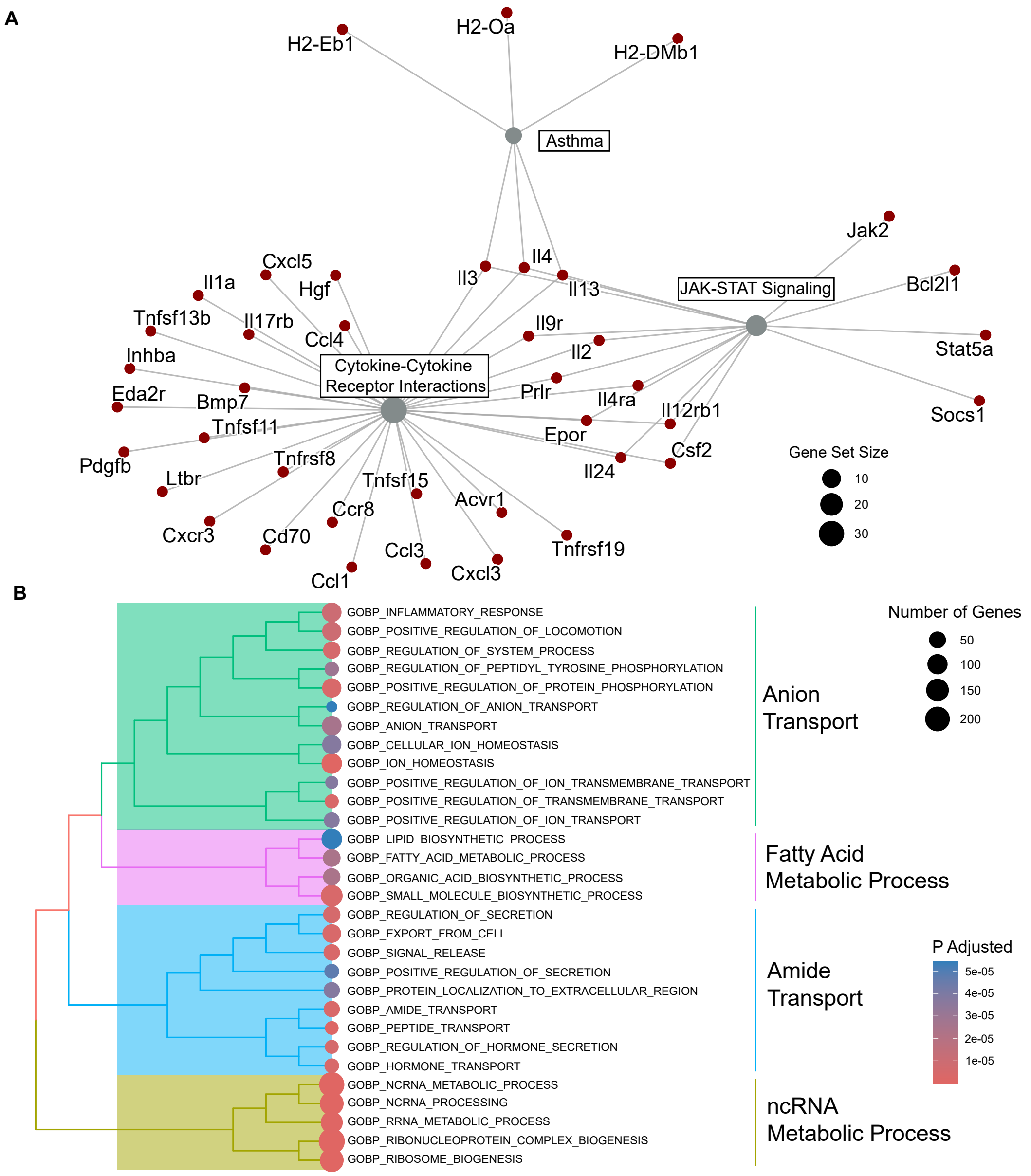

Fig. S8. Gene concept networking and tree plot analysis of differentially expressed genes in *Cftr*$^{+/+}$ vs. *Cftr*$^{-/-}$ Th2 cells. **(A)** Gene concept network highlighting connections between JAK/STAT signaling (NES =2.57, p=8.10x10^{-4}, q=6.08x10^{-4}), cytokine-cytokine receptor interaction (NES =2.95, p=1.29x10^{-7}, q=9.66x10^{-8}), and asthma (NES =2.94, p=3.19x10^{-2}, q=2.39x10^{-2}) KEGG gene sets. Grey nodes represent gene-sets, while red circles represent individual genes. The size of each grey node represents the number of genes in the gene set. NES = normalized enrichment score. p = adjusted p value. **(B)** Tree plot representing hierarchical clustering of the 30 most enriched GO:BP gene sets ranked by NES. Clustering utilized pairwise similarities of enriched terms using Jaccard's similarity index. The size of each circle represents the number of genes in that gene set. The color of the circle corresponds to the adjusted *P* value of the gene set in the enrichment analysis ($n = 3$ mice per genotype).

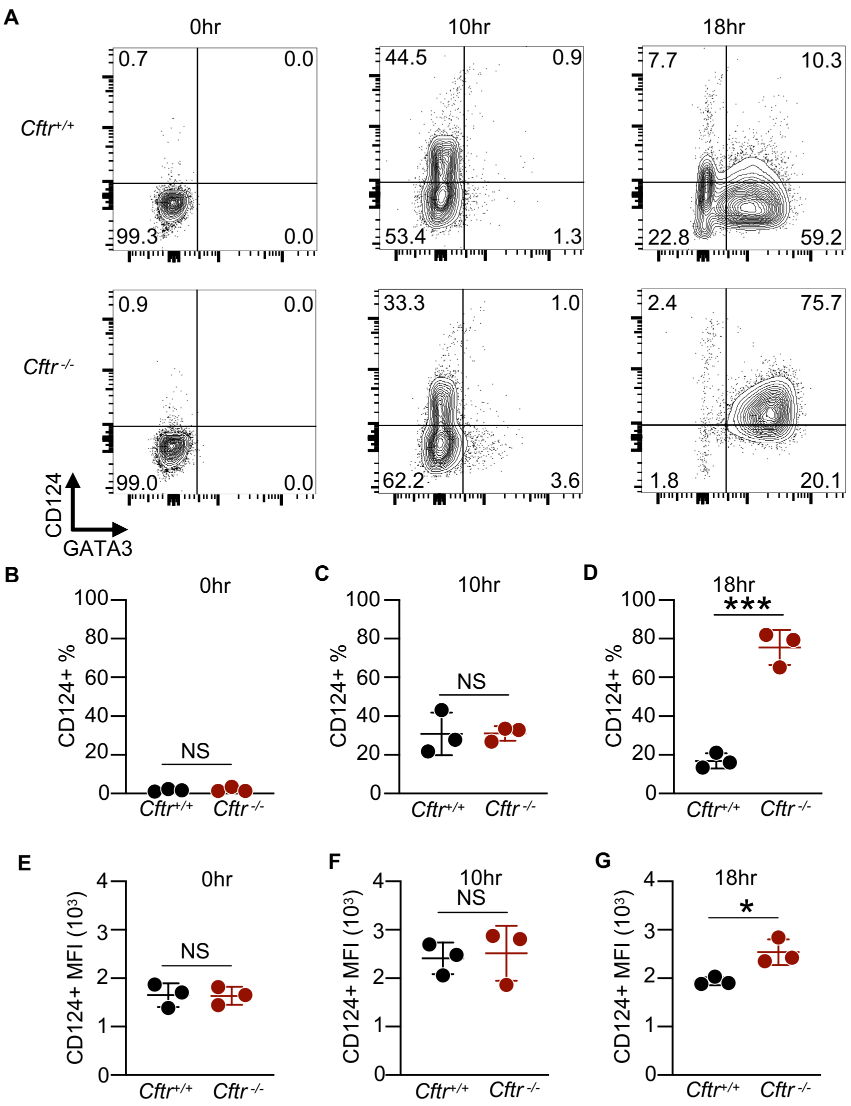

Fig. S9. IL-4Rα and GATA3 expression in *Cftr*$^{+/+}$ and *Cftr*$^{-/-}$ CD4^{+} T cells following TCR ligation and Th2 polarization. (A) Representative gating strategy for IL-4Rα (CD124) and GATA3 expression in cultured *Cftr*$^{+/+}$ and *Cftr*$^{-/-}$ CD4^{+} T cell populations gated on live cells at 0, 10, and 18 hours following activation and stimulation with mouse IL-4. (B) **(B-D)** The quantified percent IL-4Rα (CD124) positive of total cells in *Cftr*$^{+/+}$ and *Cftr*$^{-/-}$ CD4^{+} T cell populations at **(B)** 0, **(C)** 10, and **(D)** 18 hours following activation ($n = 3$ mice per genotype). **(E-G)** The IL-4Rα (CD124) median fluorescence intensity (MFI) of cultured *Cftr*$^{+/+}$ and *Cftr*$^{-/-}$ CD4^{+} T cells at **(E)** 0, **(F)** 10, and **(G)** 18 hours following activation ($n = 3$ mice per genotype). Statistical analysis (B-G) performed using unpaired student's t test, data plotted as mean ± SD. $^{*}P < 0.05$ and $^{***}P < 0.001$. NS = not significant.

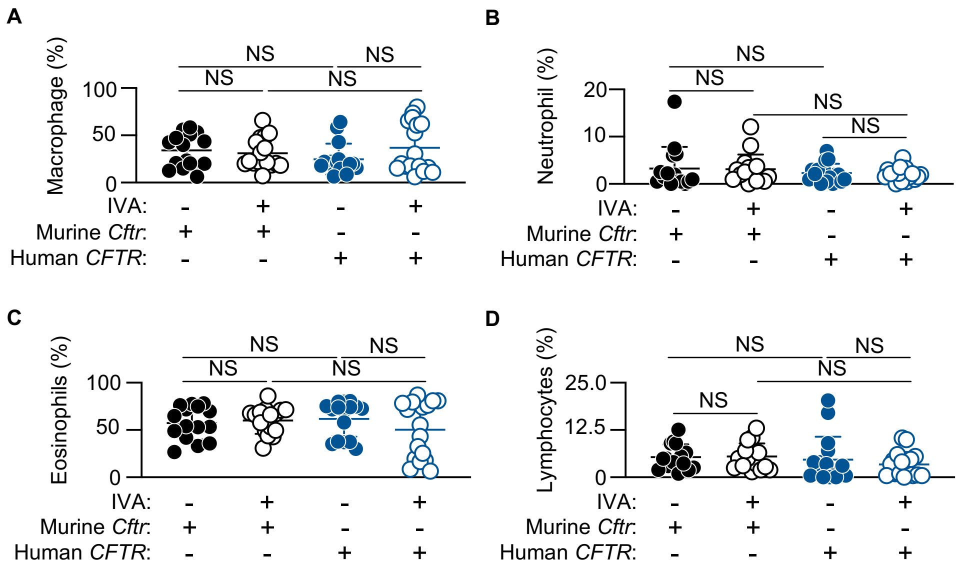

Fig. S10. Immune cell abundance in BAL from modulator treated mouse experiments. The percentages of **(A)** macrophages, **(B)** neutrophils, **(C)** eosinophils, and **(D)** lymphocytes, in the BALF of *AE*-challenged $Cftr^{+/+}$ and $Cftr^{-/-}hCFTR^{+/+}$ mice ($n = 15–17$ per genotype per condition). Open circles represent *AE* sensitized and challenged mice and closed circles denote PBS control mice. Statistical analysis in (A-H) by one-way ANOVA followed by Tukey's honestly significant difference (HSD) post hoc test for multiple comparisons. NS = not significant.

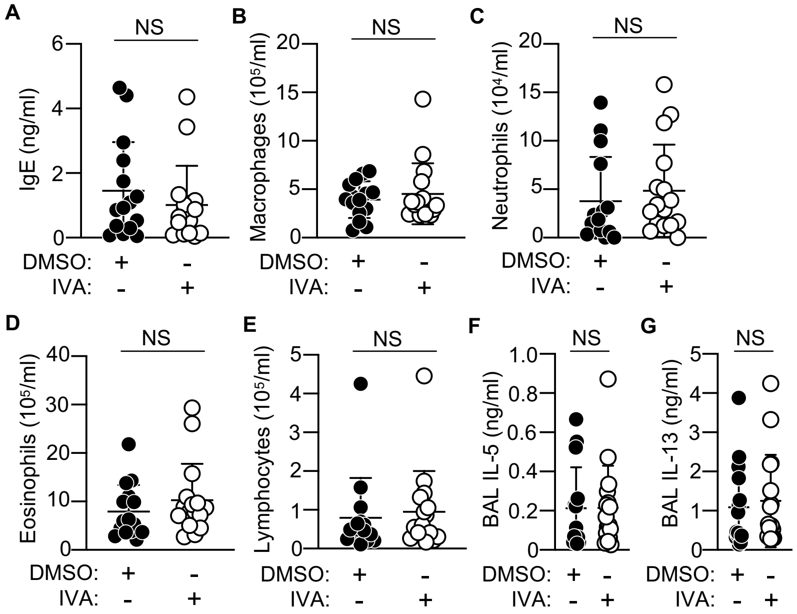

Fig. S11. Ivacaftor treatment does not decrease allergic inflammation in mice lacking human CFTR. (A) IgE concentrations by ELISA in serum from sensitized/challenged mice treated with ivacaftor (IVA, *n* =16) or DMSO (*n* =15). The number of **(B)** macrophages, **(C)** neutrophils, **(D)** eosinophils, and **(E)** lymphocytes, in the BALF of *AE*-challenged mice treated with either IVA (*n* =16) or DMSO (*n* =15). **(G)** IL-5 and **(H)** IL-13 by ELISA in BAL from *AE*-sensitized and challenged mice treated with either IVA (*n* =16) or DMSO (*n* =15). Open circles represent IVA treated mice and closed circles denote DMSO treated control mice. Statistical analysis in (A-G) performed using unpaired student's t test, data plotted as mean ± SD. NS = not significant.

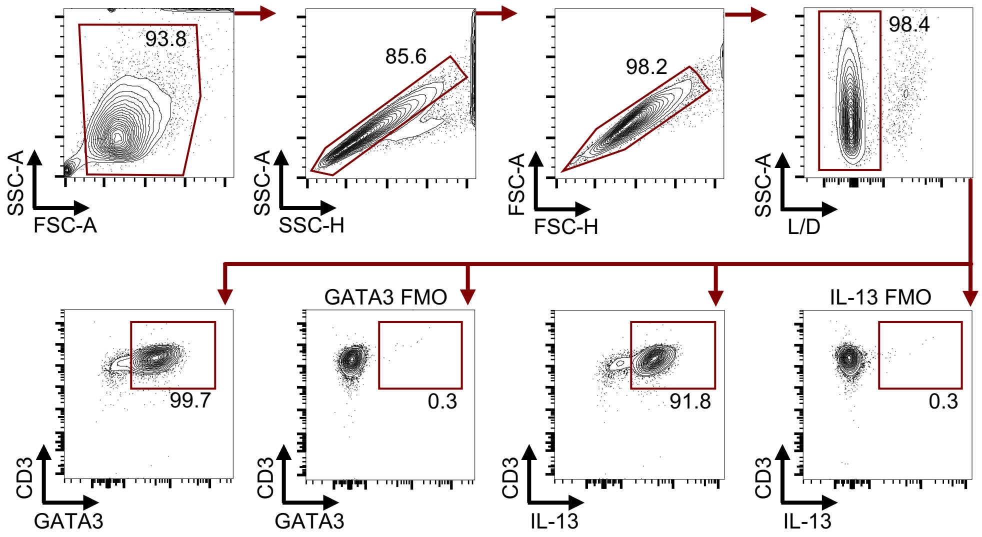

Fig. S12. Representative gating strategy for cultured human CD4$^+$ T cells. Representative gating strategy and dot plot for intracellular cytokine and transcription factor analytical flow cytometry analysis of magnetic bead enriched human CD62L^{hi}CD44^{lo}CD4$^+$ T cells cultured in Th2 polarizing conditions. LIVE/DEAD Dead Cell Stain (L/D).

Rank	Symbol	log2FC	log2FDR	Gene Name
1	Inhba	4.37	150	inhibin beta-A
2	Epas1	3.43	135	endothelial PAS domain protein 1
3	Tanc2	1.59	132	tetratricopeptide repeat, ankyrin repeat and coiled-coil containing 2
4	Rxra	1.53	130	retinoid X receptor alpha
5	Cpd	1.34	123	carboxypeptidase D
6	Gsn	1.75	107	gelsolin
7	Tnfsf11	1.71	106	tumor necrosis factor (ligand) superfamily, member 11
8	Mgll	2.97	100	monoglyceride lipase
9	Gask1a	2.84	97.1	golgi associated kinase 1A
10	Crabp2	1.25	86.6	cellular retinoic acid binding protein II
11	Trib1	1.34	81.7	tribbles pseudokinase 1
12	Il12rb1	1.37	79.8	interleukin 12 receptor, beta 1
13	Scd2	1.32	79.2	stearoyl-Coenzyme A desaturase 2
14	Il2	3.12	78.8	interleukin 2
15	Hipk2	1.27	71.6	homeodomain interacting protein kinase 2
16	Fads2	1.37	69.8	fatty acid desaturase 2
17	Bcl2l1	1.12	67.4	BCL2-like 1
18	Il3	3.78	66.8	interleukin 3
19	Cdc42bpa	1.81	64.5	CDC42 binding protein kinase alpha
20	Itpripl2	2.17	64.5	inositol 1,4,5-triphosphate receptor interacting protein-like 2
21	Tent5a	1.81	62	terminal nucleotidyltransferase 5A
22	Cd83	1.52	59.5	CD83 antigen
23	Efhd2	1.06	59.3	EF hand domain containing 2
24	Marcks	1.22	57.4	myristoylated alanine rich protein kinase C substrate
25	Mapre2	1.05	56.6	microtubule-associated protein, RP/EB family, member 2
26	Rai14	1.87	55.2	retinoic acid induced 14
27	Tnfrsf8	3.54	53	tumor necrosis factor receptor superfamily, member 8
28	Pdgfb	1.90	52.8	platelet derived growth factor, B polypeptide
29	Stra6	4.54	51.6	stimulated by retinoic acid gene 6
30	Plk3	1.23	48.9	polo like kinase 3
31	Tg	1.31	48.3	thyroglobulin
32	Sdc1	2.65	47.4	syndecan 1
33	Jak2	0.72	46.9	Janus kinase 2
34	Slc7a5	0.94	46	solute carrier family 7 (cationic amino acid transporter, y+ system), member 5
35	Cptp	1.39	44.8	ceramide-1-phosphate transfer protein
36	Il4	3.88	43.3	interleukin 4
37	Shmt2	0.91	42.6	serine hydroxymethyltransferase 2 (mitochondrial)
38	Myo6	1.23	42.5	myosin VI
39	Ccr8	1.36	41.6	C-C motif chemokine receptor 8
40	Stat5a	0.85	41.5	signal transducer and activator of transcription 5A
41	Serpinb6b	2.09	41	serine (or cysteine) peptidase inhibitor, clade B, member 6b
42	Batf	1.21	40.6	basic leucine zipper transcription factor, ATF-like
43	Axl	0.83	40.6	AXL receptor tyrosine kinase
44	P2ry1	1.08	40.6	purinergic receptor P2Y, G-protein coupled 1
45	Hsd17b7	1.08	40.2	hydroxysteroid (17-beta) dehydrogenase 7
46	Fkbp4	0.84	39.7	FK506 binding protein 4
47	Srm	1.10	39.6	spermidine synthase
48	Ybx3	0.76	39.4	Y box protein 3
49	Scd1	3.18	39.1	stearoyl-Coenzyme A desaturase 1
50	Ptpn5	1.33	38.3	protein tyrosine phosphatase, non-receptor type 5

Table S1. Top 50 most significant genes enriched in mouse *Cftr*$^{-/-}$ Th2 cells compared to *Cftr*$^{+/+}$ Th2 cells. Table indicates ranked genes enriched in *Cftr*$^{-/-}$ Th2 cells, $\log_2$ Fold Change (FC), and $\log_2$ adjusted p value for false discovery rate ($\log_2$FDR).

Rank	Symbol	log2FC	log2FDR	Gene Name
1	Ipcef1	-1.67	204	interaction protein for cytohesin exchange factors 1
2	Angptl2	-2.05	169	angiopoietin-like 2
3	Synpo	-2.32	147	synaptopodin
4	Gimap4	-1.67	138	GTPase, IMAP family member 4
5	Myb	-1.77	137	myeloblastosis oncogene
6	Gvin-ps7	-1.42	128	NA
7	Adamts6	-2.11	122	ADAM metallopeptidase with thrombospondin type 1 motif 6
8	Kbtbd11	-1.45	118	kelch repeat and BTB (POZ) domain containing 11
9	Dgka	-1.16	106	diacylglycerol kinase, alpha
10	Trp53inp1	-1.46	103	transformation related protein 53 inducible nuclear protein 1
11	Btg1	-1.32	102	BTG anti-proliferation factor 1
12	Gbp7	-2.03	100	guanylate binding protein 7
13	Klf2	-2.21	98	Kruppel-like transcription factor 2 (lung)
14	Ephx1	-1.73	89.6	epoxide hydrolase 1, microsomal
15	Slc29a1	-1.41	88.6	solute carrier family 29 (nucleoside transporters), member 1
16	Lbh	-1.56	85.4	limb-bud and heart
17	Slfn1	-1.78	81.4	schlafen 1
18	Nfkbiz	-1.55	81.3	nuclear factor of kappa light polypeptide gene enhancer in B cells inhibitor
19	Snn	-1.94	77.7	stannin
20	Ifi209	-2.47	77.3	interferon activated gene 209
21	St6gal1	-1.34	76	beta galactoside alpha 2,6 sialyltransferase 1
22	Gbp5	-1.98	75.1	guanylate binding protein 5
23	Sla	-1.10	74	src-like adaptor
24	Igf1r	-1.07	72.8	insulin-like growth factor I receptor
25	Aff3	-1.13	71.9	AF4/FMR2 family, member 3
26	Bcl2l11	-1.41	71.7	BCL2 like 11
27	Gvin2	-2.01	69.8	GTPase, very large interferon inducible, family member 2
28	Gimap3	-0.99	68.5	GTPase, IMAP family member 3
29	Cd27	-1.43	67	CD27 antigen
30	Ltb	-1.30	66.7	lymphotoxin B
31	Arel1	-1.13	66.1	apoptosis resistant E3 ubiquitin protein ligase 1
32	Pitpnc1	-1.34	65.9	phosphatidylinositol transfer protein, cytoplasmic 1
33	Gbp2	-1.77	65.8	guanylate binding protein 2
34	Gnaq	-1.66	65.7	guanine nucleotide binding protein, alpha q polypeptide
35	Smim36	-1.56	65.4	small integral membrane protein 36
36	Anxa2	-1.37	62.6	annexin A2
37	Qser1	-1.28	62.3	glutamine and serine rich 1
38	Mmd	-1.06	61.6	monocyte to macrophage differentiation-associated
39	Gimap6	-1.05	61.5	GTPase, IMAP family member 6
40	Hif1a	-1.03	59.4	hypoxia inducible factor 1, alpha subunit
41	Igfbp7	-1.82	59.3	insulin-like growth factor binding protein 7
42	Ripor2	-1.26	59.1	RHO family interacting cell polarization regulator 2
43	Cd3e	-0.88	58.3	CD3 antigen, epsilon polypeptide
44	Vsir	-1.09	57.6	V-set immunoregulatory receptor
45	Trib2	-1.38	56.7	tribbles pseudokinase 2
46	Arhgef9	-1.65	56.2	CDC42 guanine nucleotide exchange factor 9
47	Ms4a4b	-2.21	56.1	membrane-spanning 4-domains, subfamily A, member 4B
48	Ms4a6b	-0.92	53.2	membrane-spanning 4-domains, subfamily A, member 6B
49	Pecam1	-1.35	53.2	platelet/endothelial cell adhesion molecule 1
50	Ubash3b	-1.07	53.2	ubiquitin associated and SH3 domain containing, B

Table S2. Top 50 most significant genes enriched in mouse *Cftr*$^{+/+}$ Th2 cells compared to *Cftr*$^{-/-}$ Th2 cells. Table indicates ranked genes enriched in *Cftr*$^{+/+}$ Th2 cells, $\log_2$ Fold Change (FC), and $\log_2$ adjusted p value for false discovery rate ($\log_2$FDR).

KEGG Gene Set	Size	NES	FDR q-Val
Cytokine-cytokine receptor interaction	116	0.6319	5.2e-07
IL-4/IL-17 signaling pathway	183	0.7225	2.3e-05
Ribosome biogenesis in eukaryotes	90	0.6349	1.5e-04
Rheumatoid arthritis	323	0.6828	2.4e-04
PPAR signaling pathway	102	0.6883	1.4e-03
Proteasome	99	0.667	1.5e-03
Amoebiasis	276	0.6275	4.3e-03
Aminoacyl-tRNA biosynthesis	72	0.6549	4.6e-03
Arginine and proline metabolism	24	0.703	5.2e-03
Glycine serine and threonine metabolism	20	0.7128	1.3e-02
Asthma	319	0.7812	1.6e-02
Fat digestion and absorption	249	0.7241	4.3e-02
ABC transporters	89	0.6327	5.7e-02
Complement and coagulation cascades	170	0.6335	6.5e-02
Biosynthesis of unsaturated fatty acids	76	0.6458	7.0e-02
Steroid biosynthesis	11	0.7043	7.1e-02
Fatty acid elongation	9	0.6422	8.7e-02
Proximal tubule bicarbonate reclamation	242	0.7076	8.8e-02
Phenylalanine metabolism	27	0.7831	9.3e-02
2-Oxocarboxylic acid metabolism	79	0.6876	9.3e-02

Table S3. Top gene sets enriched in mouse *Cftr*$^{-/-}$ Th2 cells compared to *Cftr*$^{+/+}$ Th2 cells. Table indicates Kyoto Encyclopedia of Genes and Genomes (KEGG) gene sets enriched in *Cftr*$^{-/-}$ Th2 cells, number of genes included in gene set (size), normalized enrichment score (NES), and adjusted p value for false discovery rate (FDR q-Val). PPAR, Peroxisome proliferator-activated receptor; ABC, ATP-binding cassette.
