# Supplementary material for: CFTR negatively reprograms Th2 cell responses, and CFTR potentiation restrains allergic airway inflammation
Source: JCI Insight. 2025 Mar 25;10(9):e191098. doi: 10.1172/jci.insight.191098 (PMC12128969; doi:10.1172/jci.insight.191098)
Supplement: Unedited blot and gel images [file jciinsight-10-191098-s258.pdf]

Figure S1. Original images details

PCR products were analyzed on 2% Agarose gel, stained with SYBR Green nucleic acid dye (Invitrogen) and visualized under UV light. Western blot mages were obtained using the LI-COR Odyssey CLx imaging system. Headings above the figures correspond to the figure panels these blots were used for. Images represent the actual signal and used for the manuscript figures.

Full Gel for Figure 1A

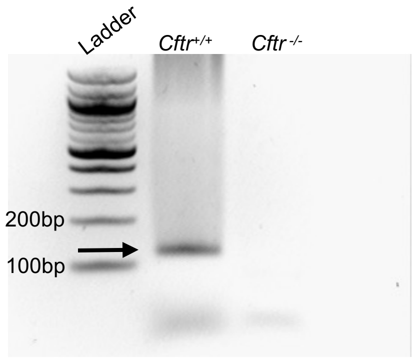

Fig S1.1. Full agarose gel electrophoresis of *Cftr* reverse-transcriptase PCR products from *Cftr*$^{+/+}$ and *Cftr*$^{-/-}$ CD4^{+} T cells.

Full blot for Figure 1D

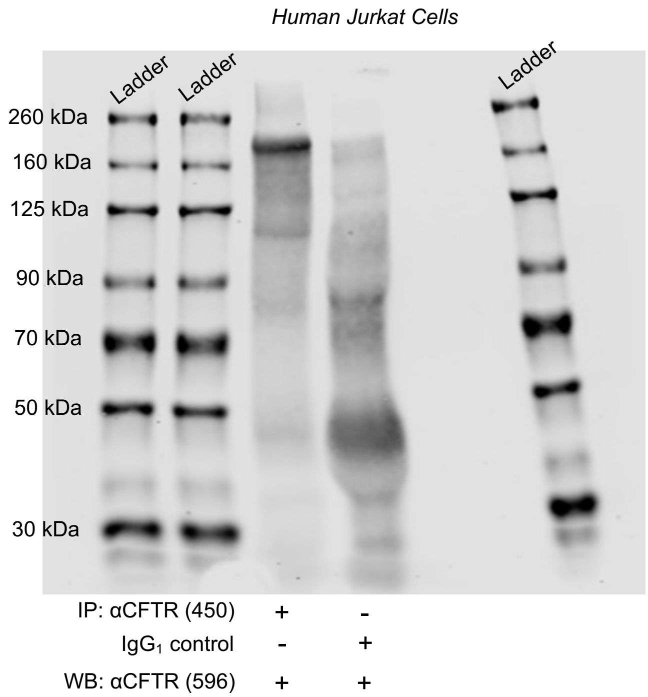

Fig. S1.2. The full image of western blot with markered IP conditions and ladders corresponding to Figure 1D.
